# Deviation from baseline mutation burden provides powerful and robust rare-variants association test for complex diseases

**DOI:** 10.1093/nar/gkab1234

**Published:** 2021-12-20

**Authors:** Lin Jiang, Hui Jiang, Sheng Dai, Ying Chen, Youqiang Song, Clara Sze-Man Tang, Shirley Yin-Yu Pang, Shu-Leong Ho, Binbin Wang, Maria-Mercedes Garcia-Barcelo, Paul Kwong-Hang Tam, Stacey S Cherny, Mulin Jun Li, Pak Chung Sham, Miaoxin Li

**Affiliations:** Program in Bioinformatics, Zhongshan School of Medicine and The Fifth Affiliated Hospital, Sun Yat-sen University, Guangzhou, China; Research Center of Medical Sciences, Guangdong Provincial People's Hospital, Guangdong Academy of Medical Sciences, Guangzhou, China; Key Laboratory of Tropical Disease Control (Sun Yat-sen University), Ministry of Education, Sun Yat-sen University, Guangzhou, China; Center for Precision Medicine, Sun Yat-sen University, Guangzhou, China; Program in Bioinformatics, Zhongshan School of Medicine and The Fifth Affiliated Hospital, Sun Yat-sen University, Guangzhou, China; Key Laboratory of Tropical Disease Control (Sun Yat-sen University), Ministry of Education, Sun Yat-sen University, Guangzhou, China; Center for Precision Medicine, Sun Yat-sen University, Guangzhou, China; Program in Bioinformatics, Zhongshan School of Medicine and The Fifth Affiliated Hospital, Sun Yat-sen University, Guangzhou, China; Key Laboratory of Tropical Disease Control (Sun Yat-sen University), Ministry of Education, Sun Yat-sen University, Guangzhou, China; Center for Precision Medicine, Sun Yat-sen University, Guangzhou, China; Program in Bioinformatics, Zhongshan School of Medicine and The Fifth Affiliated Hospital, Sun Yat-sen University, Guangzhou, China; Key Laboratory of Tropical Disease Control (Sun Yat-sen University), Ministry of Education, Sun Yat-sen University, Guangzhou, China; Center for Precision Medicine, Sun Yat-sen University, Guangzhou, China; School of Biomedical Sciences, the University of Hong Kong, Hong Kong, SAR China; State Key Laboratory of Brain and Cognitive Sciences, the University of Hong Kong, Hong Kong, SAR China; Department of Surgery, the University of Hong Kong, Hong Kong, SAR China; Dr. Li Dak-Sum Research Centre, The University of Hong Kong – Karolinska Institutet Collaboration in Regenerative Medicine, Hong Kong, SAR China; Division of Neurology, Department of Medicine, the University of Hong Kong, Hong Kong, SAR China; Division of Neurology, Department of Medicine, the University of Hong Kong, Hong Kong, SAR China; Department of Genetics, National Research Institute for Family Planning, Beijing, China; Department of Surgery, the University of Hong Kong, Hong Kong, SAR China; Department of Surgery, the University of Hong Kong, Hong Kong, SAR China; Dr. Li Dak-Sum Research Centre, The University of Hong Kong – Karolinska Institutet Collaboration in Regenerative Medicine, Hong Kong, SAR China; Faculty of Medicine, Macau University of Science and Technology, Macau, SAR China; School of Public Health, Tel Aviv University, Israel; The Province and Ministry Co-sponsored Collaborative Innovation Center for Medical Epigenetics, Tianjin Medical University, Tianjin 300070, China; The Centre for PanorOmic Sciences, the University of Hong Kong, Hong Kong, SAR China; State Key Laboratory of Brain and Cognitive Sciences, the University of Hong Kong, Hong Kong, SAR China; Department of Psychiatry, the University of Hong Kong, Hong Kong, SAR China; Program in Bioinformatics, Zhongshan School of Medicine and The Fifth Affiliated Hospital, Sun Yat-sen University, Guangzhou, China; Key Laboratory of Tropical Disease Control (Sun Yat-sen University), Ministry of Education, Sun Yat-sen University, Guangzhou, China; Center for Precision Medicine, Sun Yat-sen University, Guangzhou, China; The Centre for PanorOmic Sciences, the University of Hong Kong, Hong Kong, SAR China; Guangdong Provincial Key Laboratory of Biomedical Imaging and Guangdong Provincial Engineering Research Center of Molecular Imaging, The Fifth Affiliated Hospital, Sun Yat-sen University, Zhuhai, China

## Abstract

Identifying rare variants that contribute to complex diseases is challenging because of the low statistical power in current tests comparing cases with controls. Here, we propose a novel and powerful rare variants association test based on the deviation of the observed mutation burden of a gene in cases from a baseline predicted by a weighted recursive truncated negative-binomial regression (RUNNER) on genomic features available from public data. Simulation studies show that RUNNER is substantially more powerful than state-of-the-art rare variant association tests and has reasonable type 1 error rates even for stratified populations or in small samples. Applied to real case-control data, RUNNER recapitulates known genes of Hirschsprung disease and Alzheimer's disease missed by current methods and detects promising new candidate genes for both disorders. In a case-only study, RUNNER successfully detected a known causal gene of amyotrophic lateral sclerosis. The present study provides a powerful and robust method to identify susceptibility genes with rare risk variants for complex diseases.

## INTRODUCTION

Rare genetic variants, typically defined as variants with a minor allele frequency <1% in the population, play an important role in the etiology of complex diseases (1–3). They may explain a nontrivial fraction of ‘missing heritability’ reported by genome-wide association studies based on common genetic variants (4). Recent advances in high throughput sequencing (HTS) technologies have made it feasible to perform genome-wide association analyses of rare genetic variants in large samples. For example, the UK Biobank exomes project highlighted the significant contribution of rare variants to common diseases (5). The UK10K project has uncovered highly penetrant rare variants for cardiometabolic traits (6), and the Trans-Omics for Precision Medicine (TopMed) project will examine contributions of rare variants to heart, lung, blood and sleep disorders (7). Rare susceptibility variants have also been linked to many chronic diseases, including type 2 diabetes, myocardial infarction, osteoporosis, and Alzheimer's disease (3). Compared to common variants, rare variants are more likely to be evolutionarily new and deleterious. They are also more likely to have a larger effect on disease risk and thus provide a stronger basis for discovering new biological mechanisms or drug targets, e.g. the lipoprotein pathway gene *PCSK9* for low-density lipoprotein cholesterol levels (8).

Despite these promising early findings, studies on rare susceptibility variants suffer from low statistical power (2). As testing rare variant association at a single site generally has little power, most current methods aggregate all rare variants within a gene or genomic region to test for association with a disease (2). Such tests typically compare rare variant burden between patients and controls in terms of the mean (9,10) and variance (11) of rare variant counts. Despite the gain in power over single variant tests, existing aggregate-based association tests still require very large samples (12), which are difficult and costly to collect. To increase study efficiency, researchers have proposed to use publicly available sequencing data to boost the number of controls (13,14). However, since rare variants exhibit greater population differences than common variants (15), the use of publicly shared controls may exacerbate the challenge of hidden population stratification, leading to more spurious associations.

Here, we propose a novel statistical method to detect rare susceptibility variants in genes or genomic regions. The proposed method uses a novel strategy for association testing, which models the baseline mutation burden across genes by considering multiple genomic features as predictors and then evaluates the deviation of the observed mutation burden in a particular gene against its predicted baseline value. After characterizing the type 1 error rate, power, and robustness to population stratification of the method using simulated data, we applied it to real data sets to examine its ability to detect disease-related associations.

## MATERIALS AND METHODS

### The proposed recursive truncated negative-binomial regression (RUNNER)

#### The truncated negative-binomial regression model

We extended the negative-binomial regression model to evaluate the burden for revealing potential susceptibility genes or loci of complex diseases, assuming that a gene with excessive rare mutation burden in patients is more likely to confer disease risk. The burden is measured by an excess of observed mutation counts relative to expected baseline mutation counts. Let a gene *i* have ${m_i}$ rare variants (including single nucleotide variants and InDels with allele frequency ≤1% in reference populations) in sequenced patients. Preferably, when there are control samples, one can further remove variants with *d* (>1) times smaller allele frequency in patients than in controls. The *d* approximately corresponds to the effect size of rare variants. As the odds ratio of most rare variants of complex diseases is over 2 (16), we recommend a 2 or larger value for *d*. The *d* helps remove variants with high minor allele frequency in a local sample but rare in a reference population and leads to the baseline estimation specifically among subset variants with higher frequency in patients. Let the rare variant *j* of gene *i* have *n_i,j_* (*n_i,j_*= 0, 1, 2, …) minor alleles among all patients. Assume an integer weight (${w_{i,j}}$) can further amplify the impact of minor alleles, i.e., ${c_{i,j}} = {n_{i,j}}{\rm{\ }} \times {w_{i,j}}$. The integer weight is converted from a bioinformatics prediction score (see details in the following section), ${s_{i,j}} \in [ {0,1} ]$ by the least integer function,$\ \ {w_{i,j}} = \ [ {\frac{{{{\rm{s}}_{i,j}}}}{b}} ]$. The $b \in ( {0,1} ]$ is a score bin length parameter to determine the weight scale (See details in the below grid-search algorithm). A special scenario is that all variants has equal weight 1. According to our previous study on cancer somatic mtations (17), the total number of weighted rare minor allele counts in gene *i*, *y_i_*, can be assumed to approximately follow a negative binomial (NB) distribution:

(1) $$\begin{equation*}{y_i} = \mathop \sum \limits_{j = 1}^{{m_i}} {c_{i,j}}\ \sim NB\left( {{\mu _i},\theta } \right),\end{equation*}$$

where ${\mu _i}$ is the expected number of minor allele count in gene *i*, and $\theta$ is a dispersion parameter. The probability mass function (PMF) of NB distribution is $f ( {{y_i}|{\mu _i},\theta } ) = \frac{{{\rm{\Gamma }}( {{y_i} + \theta } )}}{{{\rm{\Gamma }}( \theta ){y_i}!}}\ \frac{{\mu _i^{{y_i}}{\theta ^\theta }}}{{{{( {{\mu _i} + \theta } )}^{{y_i} + \theta }}}}$, where ${\rm{\Gamma }}()$ is the gamma function and ${y_i}$= 0, 1, 2, ….

**Figure 1.**
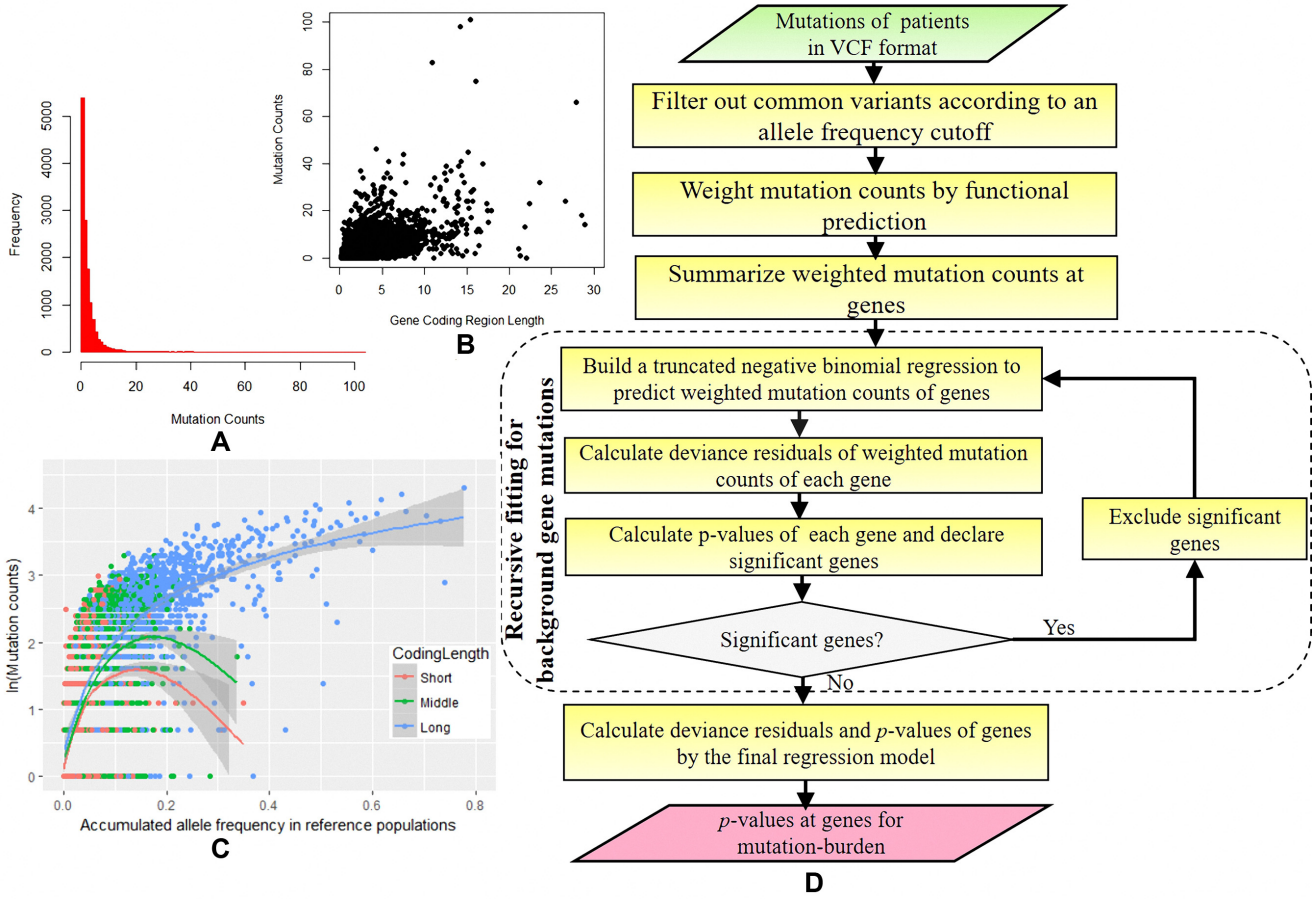
The distribution features of genes' rare mutation counts and the proposed framework. (**A**) The histogram of rare mutation counts. (**B**) The scatter plot of rare mutation counts of genes with different coding region length. (**C**) The relationship between mutation counts and accumulated allele frequency in genes with short (<1.5 kb), middle and long (>3 kb) coding region length. (**D**) The diagram of RUNNER procedure. The 493 sequenced controls of Hirschsprung disease were used to make the plots A, B and C.

However, in a sample of typical size, many genes may have no rare variants. As a result, there would be an inflation of genes with zero or a low number of minor alleles (Figure 1A), which distorts the NB distribution. Therefore, we propose to model the mutation counts by a truncated negative binomial distribution. The PMF of the distribution with a truncated point parameter *t* is:

(2) $$\begin{eqnarray*} && g \left( {{y_i}|{\mu _i},\theta ,t} \right) = \frac{{f({y_i}|{\mu _i},\theta )}}{{1 - \mathop \sum \nolimits_{j = 0}^t f(j|{\mu _i},\theta )}}, \nonumber \\ && {y_i} = {\rm{}}t + 1,{\rm{ }}t + 2, \cdots . \end{eqnarray*}$$

Meanwhile, the number of minor allele counts in a gene is affected by multiple local factors. For instance, a long gene (say *TTN*, ∼305 kb) tends to have more minor alleles than a short gene (Say *GJB1*, ∼10 kb). In the present study, we considered eight predictive factors at coding regions of genes to estimate the expected minor allele counts,

(3) $$\begin{eqnarray*}\begin{array}{@{}*{1}{l}@{}} {\eta {=} \log \left( {{\mu _i}} \right){\rm{ }} {=} {\beta _0} {+} {\beta _1} \times \left[ {{x_{1,{ i}}},{\rm{\ length\ of\ coding\ regions}}} \right] {+} {\beta _2} \times \left[ {{x_{2,{ i}}},{\rm{\ frequency\ score}}} \right] {+} {\beta _3} \times }\\ {\left[ {{x_{3,{ i}}},{\rm{\ length\ of\ coding\ regions}} \times {\rm{\ frequency\ score}}} \right] + {\beta _4} \times }\\ {\left[ {{x_{4,{ i}}},{\rm{\ observed\ over\ expected\ ratio\ for\ missense\ variants}}} \right] + {\beta _5} \times }\\ {\left[ {{x_{5,{ i}}},{\rm{\ mutation\ rate\ summed\ across\ all\ possible\ missense\ variants\ }}} \right] + {\beta _6} \times }\\ {\left[ {{x_{6,{ i}}},{\rm{\ observed\ over\ expected\ ratio\ for\ LOF\ variants}}} \right] + {\beta _7} \times }\\ {\left[ {{x_{7,{ i}}},{\rm{\ mutation\ rate\ summed\ across\ all\ possible\ LOF\ variants\ }}} \right] + {\beta _8} \times \left[ {{x_{8,{ i}}},{\rm{\ GC\ content\ }}} \right].} \end{array} \nonumber \\ \end{eqnarray*}$$

The frequency score, *x*_2_, is produced by accumulating allelic frequencies of the same types of rare variants in a reference database (e.g. 1000 Genomes Project or gnomAD) after matching allele-frequency and ancestry of the same gene (see details below). A gene with a higher frequency score tends to have more rare mutations. The interaction factor, *x_3_*, is motivated by observing that genes of different coding region lengths may show different correlations between the minor allele counts and allele frequencies (Figure 1C). The fourth to seventh predictors are genes’ observed over expected ratios and mutation rates for missense and loss-of-function (LOF) variants in canonical transcripts from gnomAD (18,19). A gene with a higher ratio and mutation rate also tends to have more rare mutations. The eighth predictor is the GC content of exon regions because the mutation rate tends to be higher in the GC-rich areas (20).

#### Optimization of parameters

The regression coefficients (${\beta _0}, \cdots ,{\beta _8}$) and dispersion parameter ($\theta$) are estimated by maximum likelihood approaches under the truncated negative binomial distribution in the above model. We derived the mathematical formulas of the first derivatives of the log-likelihood function, $l\ ({y_i}|{\mu _i},\theta ,t) = \ln [ {g( {{y_i}|{\mu _i},\theta ,t} )} ]\ = {l_i}$, for the maximum likelihood estimation (See details in the Supplementary Methods). We extended the source codes of the zero-truncated negative-binomial regression model in the ‘countreg’ R package (https://r-forge.r-project.org/R/?group_id=522) to estimate the above parameters. The Broyden–Fletcher–Goldfarb–Shanno (BFGS) algorithm optimized the estimation iteratively. Because RUNNER only performed the parameter estimation once for all genes in the entire genome without permutation, its expected runtime is insensitive to sample size (Supplementary Table S1). Thus, the parameter estimation can be finished in several minutes for an exome sequencing dataset.

When fitting the coefficients, the score bin length *b* and truncation point *t* are constant. Given *b*, *t*, and the fitted coefficients, the expected mutation count of gene *i* was estimated by:

(4) $$\begin{equation*}{\hat{\mu }_i} = {e^{{{\hat{\beta }}_0} + {{\hat{\beta }}_1}{x_{i,1}} + \ldots + {{\hat{\beta }}_8}{x_{i,8}}}}\ .\end{equation*}$$

In the truncated negative binomial model, the deviance residual of the model at gene *i* is:

(5) $$\begin{eqnarray*} {{e}_i} &=& {\rm{ }}sign\left( {{y_i} - {{\hat{\mu }}_i}} \right) \nonumber \\ &&\times\, \sqrt {2 \times |l\left( {{y_i}|\mu _i^*,\hat{\theta },t} \right) - l({y_i}|{{\hat{\mu }}_i},\hat{\theta },t)|} , \end{eqnarray*}$$

where $sign( x )$ is the sign function of the raw residual, ${\rm{t}}$he $\mu _i^*$ is the estimated mean given the observed count ${y_i}$ and estimated $\hat{\theta }$ in a saturated model (See details in the Supplementary Methods).

The deviance residuals are further standardized by the estimated mean ${\hat{\mu }_{e,i}}$ and standard deviation ${\hat{\sigma }_{e,i}}$,

(6) $$\begin{equation*}{{\acute{e}_i}} = \frac{{{e_i} - {{\hat{\mu }}_{e,i}}}}{{{{\hat{\sigma }}_{e,i}}}}.\end{equation*}$$

The standard normal distribution is used to approximate the corresponding *P*-value of ${{\acute{e}_i}}$:

(7) $$\begin{equation*} {p_i} = 1 - \Phi \left( {{\acute{e}_i}} \right), \end{equation*}$$

where $\Phi ( x )$ is the cumulative distribution function of the standard normal distribution. Using simulated data, we showed that the truncation at negative binomial distribution led to an approximately uniform distribution of the *P*-values (Supplementary Figure S1).

We built a grid-search algorithm to explore the optimal values of *b* and *t* under their reasonable ranges. The bin length *b* is assumed to range from 0.05 to 1. The truncation point *t* is assumed to range from 0 to 5. The search intervals of *b* and *t* are 0.05 and 1, respectively. Therefore, the grid-search has 120 (=20 × 6) *b –* *t* combinations in total. We adopted the mean log fold change (MLFC) (21) of *P*-values to quantify the departure from the uniform distribution, ${\rm{MLFC\ }} = {\rm{\ }}( {1/{{n}}} )\mathop \sum \limits_{i\ = \ 1}^n | {{\log_2}( {\frac{{{P_{( i )}}}}{{{q_{( i )}}}}} )} |$, where *P*_(_*_i_*_)_ = *i*th smallest *P* value among *n* genes and *q*_(_*_i_*_)_ = *i*/*n*. Values of MLFC near zero indicate smaller discrepancies between observed and theoretical *p* values under the uniform distribution. A large MLFC, say 0.3, indicates a high discrepancy. The 120 combinations are ranked according to MLFC of potential background genes in ascending order and number of significant genes (false discovery rate, FDR > 0.05) in descending order respectively, denoted as (*r_j,b_*, *r_j,t_*) and *j*∈[1,120]. The optimal *b* and *t* are defined as the ones resulting in the minimal summation rank, i.e. min(*r_j,b_*+ *r_j,t_*), which balances the uniform distribution departure of *P*-values and the number of significant genes.

#### The recursive procedure

Note that the truncated negative binomial regression is proposed to model the distribution of rare mutations at background genes, i.e. the null hypotheses. However, the excessive mutations in real susceptibility genes may harm the model fitting. Therefore, we propose a recursive procedure to approximate the null hypothesis of gene mutations by removing significant and suggestively significant genes. This would also remove some outlier genes with high technical artifacts as well. The following are the main steps of the recursive regression procedure (also see the entire diagram in Figure 1D):

- Perform the truncated negative binomial regression on all genes in a genome and calculate the *P*-value of mutation burden for each gene by RUNNER.
- Exclude significant genes according to a loose *P*-value cutoff, say, FDR of 0.1.
- Re-perform the truncated negative binomial regression on the remaining genes.
- Repeat steps 2 and 3 until no genes are excluded for obtaining a converged model.
- Calculate the deviance residuals and *P*-values of all genes by the converged model.

Here is an intuitive explanation of why RUNNER’s expected mutation count strategy could be potentially more powerful than a typical case-control association strategy. Consider a hypothetical example where the prevalence of a disease is 0.01. The frequency of rare variants in a genomic region is 0.001 per individual in controls but 0.01 per individual in cases. The overall frequency of rare variants in the population is, therefore, 0.001$ \times$0.99 + 0.01$ \times$0.01 = 0.00109. In a sample size of 500 cases and 500 controls, the expected number of rare variants in the cases and controls would be 10 and 1, respectively. If these expected values were observed, then the pooled rare variant frequency would be 0.0055, while the Pearson chi-squared statistic would be approximately (10 – 5.5)^2^/5.5 + (1 – 5.5)^2^/5.5 = 7.37, producing a two-tailed *P*-value of 0.0067. However, through statistical modeling, if we could infer correctly that the population frequency of minor alleles at the rare variant in the genomic region is 0.00109, the expected number of rare variants in 500 cases would be 1.09. The probability of observing ten or more variants assuming a negative binomial distribution is 2.52E-7. Note that a prerequisite of the expected mutation count strategy is an accurate estimation of the expectation, which is the aim of the recursive truncated negative-binomial regression model.

### Preparation of functional scores at mutations

As mutations do not function equally on a gene, it is reasonable to use prior weights to amplify minor alleles with higher functional implications. We adopted our previous ensemble score model by logistic regression (22,23) to generate a combined weight with 19 prediction scores (see the tools' names and descriptions in Supplementary Table S2). The primary rationale of these predictions is that variants that are evolutionarily conserved and have large effects on physical-chemical properties and protein structure may have a higher damaging impact on a gene product (24). The posterior probability values are further normalized within the range of 0 and 1. A larger posterior probability indicates a higher functional impact of the minor allele on the gene's function. For variants with missing posterior probability, the average one in the gene is used. The procedure of generating posterior probabilities has been implemented into KGGSeq for the truncated negative binomial regression.

### Preparation of frequency scores

The above frequency score in formula (3) is created by accumulating allelic frequencies at variants in a large reference population after matching allele frequency, variant type, and population ancestry. For example, define a gene *i* with *k* rare variants in a reference sample. Let a rare variant *j* of the gene *i* has allele frequency *f_i,j_* and the above integer functional weight *w_i,j_*. The frequency score of gene *i* is then equal to:

(8) $$\begin{equation*}{{\rm{x}}_{2,i}} = \mathop \sum \limits_{j = 1}^k {f_{i,j}} \times {w_{i,j}}\ .\end{equation*}$$

When there is more than one reference sample, the averaged allele frequencies of multiple samples are used for the frequency score. In the present paper, we adopted the allele frequencies of variants from the 1000 Genomes Project and in the Genome Aggregation Database (gnomAD, V2.1) as reference samples. Alternatively, one can also use the frequency scores calculated in other reference samples and even large local control samples. For the 1000 Genomes Project, the variant sites and allelic counts (Phase3 V6) of five different ancestry panels (African, Mixed American, East Asian, European and South Asian) were downloaded at ftp://ftp.1000genomes.ebi.ac.uk/vol1/ftp/release/20130502/. For gnomAD, the variant sites and allelic frequencies of seven ancestrally different panels (East Asian, South Asian, African/African American, Latino, Finnish, Non-Finnish European, and Ashkenazi Jewish) were downloaded from http://gnomad.broadinstitute.org/downloads. In gnomAD, we only included the variants and allelic frequencies from subjects labeled as controls. The variants were mapped onto genes according to physical positions in two gene models (RefGene and GENCODE) and annotated with gene features by KGGSeq (Version 1.2) (25).

### Alternative gene-based association tests for rare mutations

We compared the performance of RUNNER with four widely-used association tests for rare mutations, which used different models to evaluate the mutation burden between cases and controls.

- Combined Multivariate and Collapsing (CMC) method (9): The CMC method is a type of direct burden test for case-control samples. The variants were divided into multiple groups according to the allele frequencies. Within a group, mutations at multiple variants were collapsed. Then, a multivariate test (Hotelling's T2 test) was employed to test whether all groups' collapsed mutations were associated with case-control status.
- The sequence kernel association test (SKAT) (11): SKAT assumed the effects of rare variants on disease risk followed an arbitrary distribution with a mean of zero and an unknown variance. The variants without effect would have zero genetic variance. A score-based variance-component test in a mixed model was then constructed to examine whether the variance was equal to zero. SKAT was further extended to improve power for detecting variants with the same or different directions, SKATO (26). For power comparison, we also assigned the same weights as RUNNER to perform the weighted version of SKAT.
- The kernel-based adaptive clustering model (KBAC) (27): KBAC considered genotypes of all rare variants in a gene as multi-site genotype vectors. The disease risk for a multi-site genotype was modeled using a mixture distribution. An adaptive weighting procedure was then used to produce each genotype's weights for their causality or susceptibility to a disease based on a known component called a kernel.

The four methods were implemented into a user-friendly tool, RVTESTS (Version 20190205) (28). For the weighted SKAT, the R package of SKAT (https://cran.r-project.org/web/packages/SKAT/, version 2.0.1) was adopted. Our high-throughput sequencing data analysis platform, KGGSeq (http://pmglab.top/kggseq/, Version 1.2, source codes, https://github.com/pmglab/KGGSeq), has integrated RVTESTS and SKAT as the third-party analysis components.

### Computer simulations investigate type 1 error and power

To keep the distribution of rare background mutations, we used a semi-simulation procedure to investigate the statistical type 1 error and power of RUNNER. We first removed variants in 3 known causal or significant genes in the Hirschsprung whole-genome sequencing sample and used all the 936 subjects for the simulation (29). We randomly drew *m* (=150, 300, 600 and 900) subjects to form a new sample with half pseudo-cases and half controls. RUNNER was used to calculate the *P*-values of genes in the new sample. The quantile-quantile (QQ) plot of −log_10_ (*P*-value) under uniform distribution was used to check the distribution of *P*-values, particularly at the tail of small *P*-values. The MLFC was also adopted to quantify the deviation of *P*-values from a uniform distribution in general (21). To investigate the statistical power, we made an artificial patient sample by randomly inserting several causal mutations (simulated according to pre-set frequencies) into the genomes of pseudo-cases. Two genes (*TCF4* and *TIE1*) were set as the targeted susceptibility genes in the simulations. Either gene was assumed to have 4–5 non-synonymous susceptibility variants (See the variants in Supplementary Table S3). Each variant had an expected frequency of 1.0–1.5% at the risk alleles in the artificial patient sample. The allele frequency at the assumed susceptibility variants in a control sample was equal to the alternative allele frequency in cases divided by a random odds ratio ranged from 2 to 5. The case-control samples were analyzed for power comparison. We produced *t* case–control samples for the association tests. Assume a test had genome-wide significant *P*-values at a tested gene in *k* samples. The power was estimated as *k*/*t* at this gene.

We also used subjects from the 1000 Genomes (1KG) Project to check how the methods were sensitive to population stratification. Using all the 5 ancestry panels (East Asian, South Asian, European, American and African), we constructed 20 population-mixed samples. We randomly drew (sampling without replacement) 66.6% of a panel's subjects to constitute pseudo-cases in each sample. The remaining 33.4% of subjects in the same panel and an equal number of subjects randomly drawn (sampling without replacement) from each of the four other panels constituted the controls. The samples were analyzed by RUNNER and four alternative methods for gene-based association at rare variants. No genes were assumed to be related to the case-control membership, mimicking the null hypothesis. Thus, all significant associations would be spurious, mainly due to population stratification.

The third sample we used for the semi-simulation-based power comparison contained 4810 whole-genome-sequenced subjects from the Singapore 10K (SG10K) Genome Project (30). we conducted a down-sampling analysis in the SG10K dataset to investigate how the sample size influences the detection of significant genes discovered in a larger sample. Ten artificial susceptibility genes with 30 rare susceptibility variants (See the variants and minor allele frequencies in Supplementary Table S4) were inserted into genomes of 2405 pseudo-patients and 2405 controls according to the pre-set allele frequencies. The subset of samples with sizes 500, 1000, 1500 and 2000 was randomly drawn and analyzed by RUNNER one by one. We repeated the down-sampling procedure ten times at each sample size to obtain an averaged number of re-discovered genes to reduce stochastic sampling errors.

### Real high-throughput sequencing datasets

Three real high-throughput sequencing datasets were used to validate the performance of the proposed method. Supplementary Table S5 summarizes the disease names and sample information. All the sequencing samples were obtained with Institutional Review Board approval in either Hong Kong or mainland China. The short reads produced by Illumina HiSeq 2000 were mapped onto the reference genome (HG19) by BWA v0.7.17 (31). The redundant reads were removed by Picard (https://broadinstitute.github.io/picard/). The sequence variants were called by GATK v3.8 (32). Stringent quality control (QC) procedure at the variants was performed on KGGSeq V1.2 (http://pmglab.top/kggseq/). The variants or genotypes failing to pass the following QC criteria were excluded: Hardy-Weinberg test *P*-value ≤ 0.001, genotyping quality Score (Phred Quality Score) <20, read depth per genotype <8, having more than four alleles, genotyping rate <90% in a sample. Supplementary Table S5 also lists the initial and retained sequence variants in each dataset.

### Detection of susceptibility genes with rare variants by the proposed methods and four alternative methods

All analyses for detecting susceptibility genes with rare variants were carried out on our KGGSeq platform (http://pmglab.top/kggseq/). After QC, common variants were then filtered out according to their alternative allele frequency (allele frequency > 0.01) in two databases, gnomAD exomes and gnomAD genomes from East Asian panels. According to two gene models (RefGene and GENCODE), the variants were mapped onto genes and annotated with gene features (missense, synonymous, splicing, etc.). In the uncommon scenario that a variant was in overlapped regions of multiple genes, the variants were assigned to each of the overlapped genes. Gene-based tests were then performed to detect the diseases' potential susceptibility genes with the rare variants. The present study considers mutation types of missense, start-loss, stop-loss, stop-gain, splicing, frameshift and non-frameshift. For the gene-based association test by the four alternative methods [CMC (9), KBAC (27), SKATO (26) and SKAT (11)], KGGSeq produced input of these tools and automatically called RVTESTS (Version 20190205) for the analysis in parallel. For the baseline mutation burden test by RUNNER, KGGSeq (Version 1.2) directly ran its Java codes to perform the analysis. The functional weights to amplify minor allele counts in RUNNER were also produced by KGGSeq.

## RESULTS

### Overview of RUNNER

Our proposed method is built on the deviation of the observed rare mutation burden at a genomic region (typically a protein-coding gene) in patients against its baseline expectation in the population (see the method's workflow in Figure 1D). A weighted recursive truncated negative-binomial regression estimates the baseline expectation based on the genomic region's characteristics. The truncated negative-binomial model was proposed to respond to two distribution features: the negative binomial distribution allows for over-dispersion of rare mutation counts compared to the Poisson distribution, and truncation allows for inflated zero or low mutation counts (Figure 1A and B). With the fitted regression model, the deviance residuals of weighted rare mutation count burden at all genes are calculated, standardized to *z*-scores, and then converted to *P*-values. Thus, RUNNER is fundamentally different from conventional rare-variants methods, e.g. CMC (9) and SKAT (11), which are based on case-control differences in rare variant counts. Intuitively, the power gain of RUNNER compared to typical case-control studies depends on the extent that the weighted recursive truncated negative binomial regression can accurately model the variation in rare mutational burden across the genome and on the size of the control sample available for conventional case-control comparison. However, unlike SKAT (11), RUNNER cannot directly adjust for confounding factors. We build a logistic regression model for a disease phenotype affected by environmental factors to calculate the residual given the environmental factors. Patients with a low residual can be precluded before RUNNER analysis. Moreover, because RUNNER essentially assesses the relative mutation burden only in patients, allele frequencies in controls will not significantly influence the test as it does for the case-control test. So, spurious association due to population stratification in a typical case-control analysis may no longer be a serious issue for RUNNER (See more illustration in the Discussion section). RUNNER was implemented in our high-throughput sequencing data analysis platform KGGSeq (Version 1.2) (22,23) (http://pmglab.top/kggseq/).

### Type 1 error of the proposed and alternative methods for genome-wide association screening

**Figure 2.**
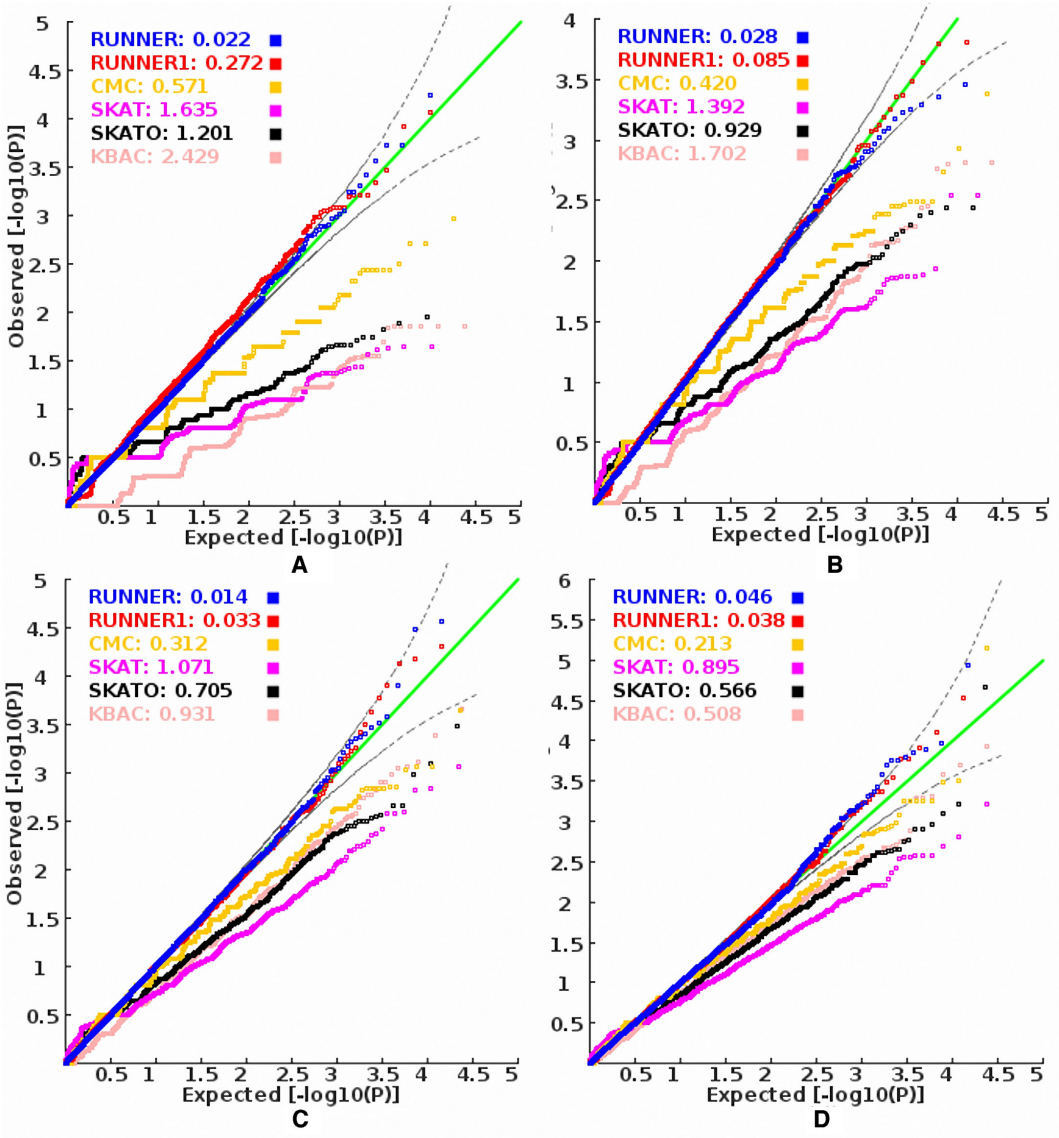
The QQ plots of *P*-values produced by different methods in random samples When the sample size of cases is (**A**) 75; (**B**) 150; (**C**) 300; (**D**) 450 and equal numbers of controls. RUNNER: the proposed approach; RUNNER1: the equal weight (weight = 1) version of RUNNER which uses original mutation counts at variants. CMC, SKAT, SKATO and KBAC are four widely-used approaches for gene-based association tests with rare variants in case-control samples. The values behind the model name denote its corresponding inflation index, MLFC.

To check whether RUNNER is valid for statistical inference, we first investigated its genome-wide statistical type 1 error for detecting susceptibility genes with rare mutations by a random sampling approach. Similar to the simulated negative binomial data (Supplementary Figure S1), the distribution of *P*-values produced by RUNNER for mutation counts in real samples was also very close to a uniform distribution *U*[0,1] (Figure 2). The uniform distribution was unchanged when the sample size was as small as 150 (half cases and half controls). The uniform distribution departure indexes, MLFC, for RUNNER were < 0.05 for all the four different sample sizes (*n* = 150, 300, 600 and 900). As expected, in the particular circumstance of equal weights (weight = 1) at variants, RUNNER1 also generated approximately uniformly distributed *P*-values. To mention it concisely, this equal weight (weight = 1) version of RUNNER is called RUNNER1 in this manuscript. In contrast, the four widely-used gene-based association methods (CMC (9), SKAT (11), SKATO (26) and KBAC (27)) for case-control analysis showed deflated *P*-value distribution when the sample size was small, say <300. The MLFC of the four methods was >0.3, indicating a severe departure from the uniform distribution. The time-consuming permutation (*n*= 10^6^) in KBAC did not rescue the deflation at small sample sizes. The result was consistent with the consensus that existing statistical association methods for rare variants had invalid approximation in their test statistics, generally in small or even moderate samples (33). The *P*-values produced by CMC deviated from uniform distribution less than that produced by three alternative methods. SKAT was more conservative than SKATO at small sample sizes. The deflation of the four methods became smaller as sample sizes increased to 900. The suitable type 1 error of RUNNER ensures a valid interpretation of RUNNER’s *P*-values for statistical inference even at small sample sizes.

We also compared the type 1 error of the truncated negative binomial model with a truncated Poisson model. The latter is also widely used for rare variant analysis, e.g. (26) (34). In a randomly drawn dataset (300 cases and 300 controls) under the null hypothesis, the generalized linear regression under the truncated Poisson distribution showed severe type 1 error inflation (Supplementary Figure S2). Many long genes (e.g. *TTN*) had very significant *P*-values. In contrast, RUNNER’s truncated negative binomial regression model showed no inflation of type 1 error in the same dataset. This comparison implied that the negative binomial model could better fit the rare mutation counts than the Poisson distribution. Probably, this was because the former can consider the overdispersion of mutation counts better than the latter.

### Robustness of RUNNER to population structure

**Figure 3.**
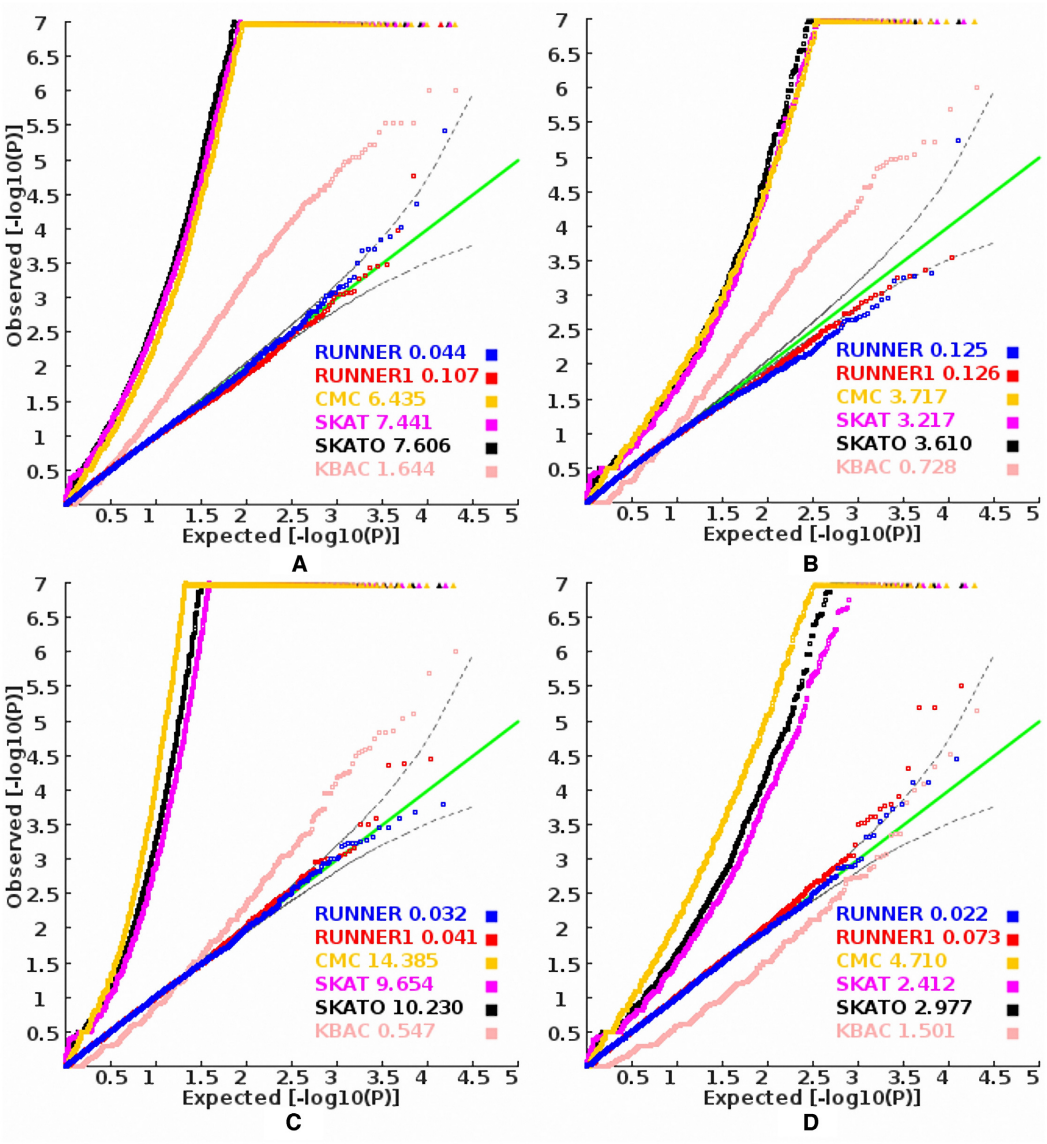
The *P*-value QQ plots of different tests in a stratified population. (**A**) African pseudo-cases vs half American controls; (**B**) American pseudo-cases vs half East Asian controls; (**C**) East Asian pseudo-cases versus half European controls and (**D**) European pseudo-cases vs American controls from the 1000 Genomes Project. The values behind the model name denote its corresponding inflation index, MLFC.

We also found RUNNER had much less type 1 error inflation to population stratification than conventional case-control association tests. It showed no inflation trend for the baseline mutation burden test in almost all 20 ancestrally admixed samples. The inflation indexes, MLFC, in all the 20 samples were less than 0.195, and there was no systematic type 1 error inflation in most samples (Figure 3 and Supplementary Figure S3). In some samples (say European pseudo-cases vs. half South Asian controls), a small fraction of genes had significant *P*-values (≤2.5E-6). Some local sample-specific common variants caused the significant *P*-values. Almost all significant *P*-values disappeared when variants with minor allele frequency ≥1.5% were excluded (Supplementary Figure S4). Similarly, the equal-weight version RUNNER1 also showed uniformly distributed *P*-values in most 20 samples (Supplementary Figure S3). In contrast, all the four alternative association tests showed inflation of type 1 error rates in most samples with 50% population stratification in controls. Figure 3 also shows their *P*-value QQ plots in four different samples in which the pseudo-patients were East Asian, European, American, and African, respectively. Over 200 genes had *P*-values < 2.5E-6 by CMC, SKAT and SKATO in all four samples. Among the four alternative methods, KBAC had less inflation than the other three, while its sensitivity to population stratification was apparent in most samples. These results suggested RUNNER can be the priority method for samples with large population stratification.

However, we noticed that it is necessary to use ancestrally matched references to filter out common variants in patient samples for RUNNER. In another artificial experiment, we investigated this issue also by using the gnomAD exomes data. As shown in Supplementary Figure S5a, when the East Asian reference population was used for pseudo-patient samples from the European panel of the gnomAD database, RUNNER showed *P*-value inflation among genes with low-frequency (MAF < 3%) variants enrichment. The issue was similar when the European reference population was used for pseudo-patient samples from the East Asian panel (Supplementary Figure S5b). With unmatched reference populations, many population-specific common variants would be retained after allele frequency-based filtration. As RUNNER is sensitive to the enrichment of mutation counts in a gene, genes having multiple population-specific common variants would have significant *P*-values. In the software platform KGGSeq, we integrated reference allele frequencies of seven ancestries from the gnomAD database (19). Although gnomAD might not provide a complete match for a local sample, the correct type 1 errors in the present paper's simulations and real applications suggested that a crude ancestry match may be acceptable for RUNNER.

### The statistical power of the proposed and alternative methods for genome-wide association screening

**Figure 4.**
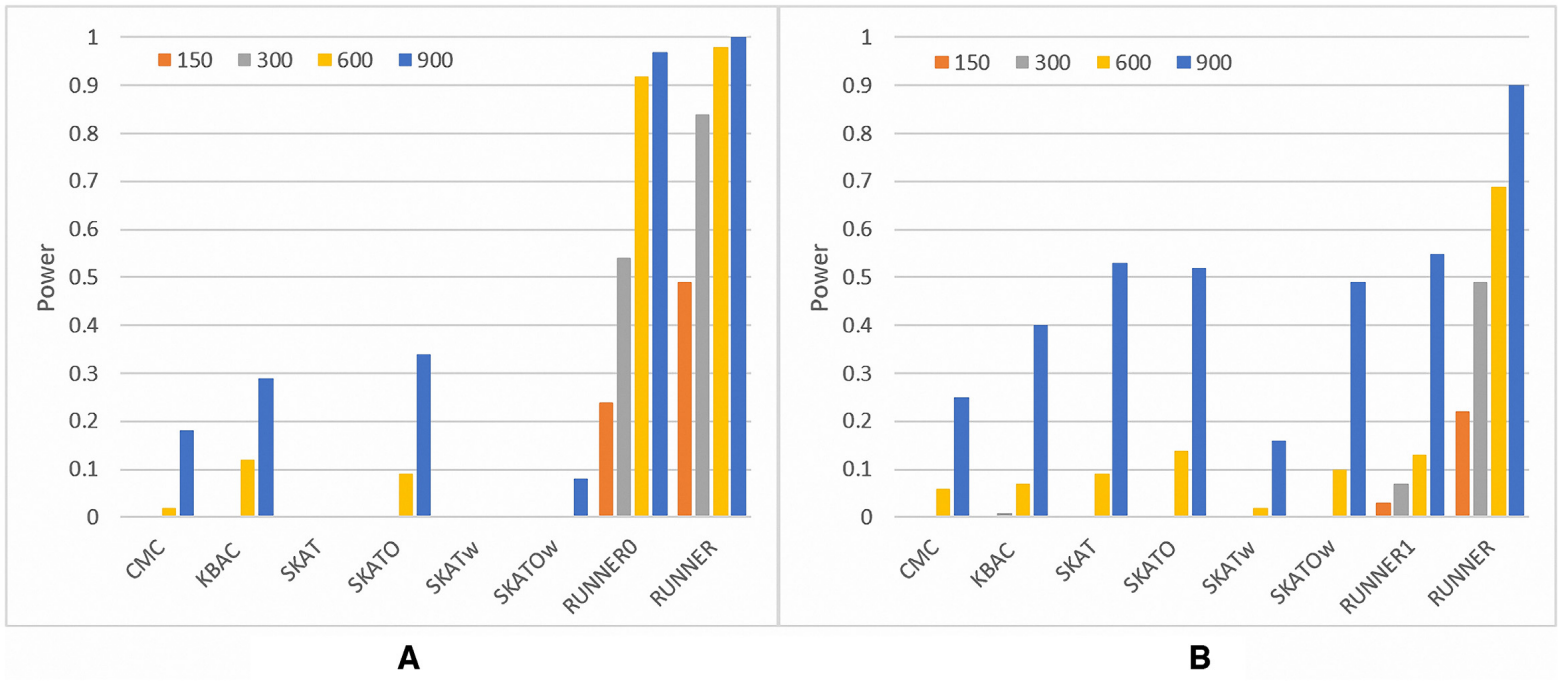
Performance comparison of different methods. (**A**) Power of methods for detecting gene *TCF4*. (**B**) Power of methods for detecting *TIE1* gene. For each scenario, 100 datasets were simulated. The assumed rare susceptibility variants can be seen in Supplementary Table S3. SKATw and SKATOw are the weighted SKAT and SKATO by using the same functional prediction scores adopted by RUNNER, respectively.

We further compared the statistical power of RUNNER with the four widely-used rare variant tests by semi-simulation analysis. The diseased samples were simulated by randomly inserting susceptibility alleles into real genomes. Multiple rare missense mutations in *TCF4* and *TIE1* genes (See the variant list in Supplementary Table S3) were assumed in the simulation. *TCF4* was repeatedly validated as a susceptibility gene for schizophrenia (35), and *TIE1* was implicated with vascular development and pathogenesis of vascular diseases (36). As shown in Figure 4, RUNNER had a much higher power to detect *TCF4* than the other four alternative methods. When the sample size was 600 (300 cases and 300 controls), RUNNER achieved 98% power while all the other four alternative methods had <35% power according to an exome-wide *P*-value cutoff of 2.5E-6. RUNNER’s power increased to 100% when the sample size increased to 900. In contrast, among the alternative methods, KBAC had the highest power (only 12%) at the sample size of 600. When the sample size increased to 900, the most powerful alternative test was SKATO, with a power of 34%. Other alternative tests had nearly zero power to detect *TCF4* with 300 cases and 300 controls. The weighted version of SKAT and SKATO even had lower power to detect this gene. This is consistent with previous studies in which existing methods for rare variants generally had very low power in small or moderate samples (12). When equal weight 1s were imposed at all variants (denoted as RUNNER1), the power of RUNNER slightly decreased, 92% and 97%, at the sample sizes of 600 and 900. Therefore, RUNNER was much more powerful than the other four alternative methods for detecting *TCF4*.

As for the other gene, *TIE1*, all methods had lower power to detect it when the sample size was <600. RUNNER had 69% higher power while the other four alternative methods had <15% at the sample size of 600. When the sample size increased to 900, RUNNER achieved 90% power while all other alternative methods had < 53% power. The higher power at *TCF4* than *TIE1* was because *TCF4* had more assumed causal mutations (5 versus 4), shorter coding region (2.83 kb versus 3.64 kb), and lower accumulated allelic frequency in reference populations. Nevertheless, RUNNER achieved the best power to detect genes with rare susceptibility mutations compared to alternative methods.

We also used the pilot study of the SG10K Genome Project (30) sample (*n*= 4810) to investigate how the sample size influenced the detection of genes significant in a large sample. Ten artificial susceptibility genes (See details in Supplementary Table S4) were set in a large sample of 2405 pseudo-cases and 2405 controls. RUNNER and RUNNER1 detected seven and five genes out of the ten, respectively (*P*≤ 2.5E-6). In sub-samples of the large sample, the number of significant genes was generally related to sample sizes (Supplementary Figure S6). For example, in a sub-sample of 500, 1000, 1500 and 2000 pseudo-cases, the weighted version of RUNNER detected 3.2, 5, 6.2 and 6.8 significant genes on average, respectively. RUNNER1 detected fewer significant genes than the weighted one at all the sub-samples.

### Validation of RUNNER in real datasets of complex diseases

We further validated the performance of RUNNER at three different complex diseases (Hirschsprung disease, Alzheimer's disease, and amyotrophic lateral sclerosis) with high throughput sequencing data (See summary description in Supplementary Table S5). Genes were tested for a rare mutation burden in patients by RUNNER. Besides, we also compared RUNNER’s results to four widely-used rare mutation association tests, including CMC (9), KBAC (27), SKAT (11) and SKATO (26).

#### Hirschsprung disease (HSCR)

HSCR, also known as congenital megacolon or intestinal aganglionosis (37), is a highly heritable disorder with significant phenotypic heterogeneity. Our dataset comprised 443 Asian HSCR patients and 493 Asian control individuals assayed by whole genome sequencing (29). According to the QQ plots (Supplementary Figure S7a), the *P*-values of RUNNER, CMC, KBAC and SKATO approximately followed the uniform distribution while SKAT showed slight deflation. Table 1 lists the top 10 genes detected by RUNNER. The well-known HSCR susceptibility gene, *RET* (38,39), was prioritized as the top gene by both RUNNER (*P*= 8.58E-8) and the equal weight version RUNNER1 (*P*= 7.01E-8). In these cases, *RET* had 61 rare nonsynonymous minor alleles (See frequencies in Supplementary Table S7). However, none of the alternative methods prioritized *RET* as a significant gene in the same set of rare variants. The top gene by CMC, SKATO, and KBAC was *OGG1* (*P*= 2.01E-6, 5.03E-6 and 1.0E-4), while SKAT ranked *ZNF276* as the top gene (*P*= 2.97E-4). But we failed to find literature to support these genes’ contribution to the development of HSCR. A more remarkable instance can be seen in *EDNRB*, another well-known HSCR susceptibility gene (39,40). *EDNRB* was the 4^th^ most significant gene by RUNNER (*P*= 2.39E-5, top 0.27‰ in all tested genes). Unfortunately, CMC, KBAC, SKAT and SKATO nearly neglected this gene, with a rank of 53, 61, 200 and 351 (top 2.2‰, 2.5‰, 8.4‰ and 14.8‰ in all tested genes), respectively. The *EDNRB* only had 15 rare nonsynonymous mutations. RUNNER1 just ranked it at 30^th^ (top 1.99‰) in all tested genes. It was the weight that boosted the significance of *EDNRB*.

**Table 1.** Top 10 genes prioritized by RUNNER in Hirschsprung disease dataset

Gene symbol	Minor alleles	Missense alleles	LOF alleles	Region length	mu_mis	mu_lof	oe_mis	oe_lof	ExonGC	Residual_1_	*P*_1_	Residual	*P*
***RET***	61	56	5	3.477	44.853	2.678	0.896	0.039	0.585	5.265	7.01E-08	5.228	8.58E-08
***NXPE4***	21	19	2	1.660	13.620	0.996	1.018	1.109	0.415	3.311	4.65E-04	4.649	1.67E-06
***CDCA4***	19	19	0	0.731	10.045	0.354	0.934	0.452	0.531	3.586	1.68E-04	4.102	2.05E-05
***EDNRB***	15	13	1	1.797	14.472	1.186	0.806	0.329	0.410	2.840	2.26E-03	4.066	2.39E-05
***OGG1***	26	20	6	1.732	13.857	1.345	1.103	0.885	0.576	3.498	2.35E-04	4.055	2.50E-05
***PHYKPL***	22	20	1	1.452	16.346	0.929	1.081	0.961	0.562	3.096	9.81E-04	3.921	4.42E-05
***GAB4***	33	32	1	2.105	18.445	0.889	1.149	0.815	0.578	3.820	6.66E-05	3.766	8.29E-05
***MOGAT2***	15	15	0	1.035	13.006	0.852	1.047	0.378	0.546	2.547	5.44E-03	3.481	2.50E-04
***KIAA1257***	31	26	5	3.452	12.522	0.811	0.896	0.871	0.481	3.823	6.58E-05	3.433	2.99E-04
***BECN1***	15	15	0	1.408	13.371	1.384	0.666	0.151	0.481	3.240	5.99E-04	3.397	3.40E-04

Notes: Minor alleles refer to rare nonsynonymous minor alleles in the tested cases. The minor allele counts to missense is summed in Missense alleles, and the minor allele counts to loss-of-function (stopgain, splicing, frameshift) is summed in LOF alleles. Region Length and ExonGC are the length (kb) and GC content in exon regions of the gene. mu_mis and mu_lof refer to the gene's mutation rate based on missense variants and loss-of-function variants in its canonical transcript, respectively. oe_mis and oe_lof refer to the gene's observed over expected ratio at missense and loss-of-function variants obtained from gnomAD, respectively. Residual_1_ and *P*_1_ denote the deviance residual and *P*-value generated by RUNNER1 (the equal-weight (weight = 1) version of RUNNER). Residual and *P* mean deviance residual and *P*-value generated by RUNNER.

Besides the two known susceptibility genes, RUNNER also detected another gene, *NXPE4*, with a significant rare mutation burden in the HSCR patients, *P*= 1.67E-6. *NXPE4* has specifically high expression in colons (see details in database REZ, http://pmglab.top/rez/) (41), and is known to be associated with ulcerative colitis (42). The high expression of *NXPE4* also had a significantly improved prognosis of colorectal cancer regarding patients' overall survival (43).

All the eight predictors in the fitted RUNNER model significantly contributed to predicting minor allele counts at background genes (Supplementary Table S6a). As expected, the coding region length and frequency scores were positively related to the rare mutation counts of genes (*P*= 8.35E-15 and 4.99E-192), which meant longer coding regions and higher mutation frequency genes tended to have more rare nonsynonymous mutations. Consistent with previous studies (20), genes with higher GC content also tended to have more rare nonsynonymous mutations (*P*= 1.68E-8). The genes’ observed over expected ratio for missense variants had a much higher significance level than for LOF variants (*P*= 1.29E-44 versus 1.69E-5). Conversely, a slightly greater contribution could be seen in the genes’ mutation rate summed on LOF variants than on missense variants (*P*= 2.03E-15 versus 4.27E-10). Interestingly, the interaction term between the coding region length and gene frequency score negatively affected the mutation counts (−0.212 ± 0.004) and had the most significant level according to its *z*-score, −53.61. The negative interaction suggested that frequency score had a smaller effect for large genes than for short genes. This meant that the genome might show local variation in mutation frequency. For a large gene, the local variation was averaged out so that the length mattered the most. For short genes, the local information was important, and the frequency score captured this. The varied tendency is visualized among genes in three gene groups of different coding region lengths (Figure 1C).

#### Alzheimer's disease (AD)

AD is a progressive neurodegenerative disorder characterized by memory loss and cognitive impairments among older people. This dataset contained 246 *APOE* ϵ4-negative Chinese AD patients and 172 healthy elderly controls sequenced at exomes (44). In this small sample, RUNNER still showed approximately uniformly-distributed *P*-values at most of the genes for the rare variant mutation burden test, while all the four alternative tests showed deflation (Supplementary Figure S7b). RUNNER1 detected a significant gene, *NAT1* (*P*= 1.24E-7), which had 27 rare nonsynonymous mutations in the AD patients (See details in Supplementary Table S8). It encodes an arylamine N-acetyltransferase, which helps function in folate catabolism. Multiple studies have suggested that low folic acid levels may be risk factors for AD (45,46). A recent study indicated that reduced expression of *NAT1* contributes to cerebral endothelial necroptosis and Aβ accumulation in AD (47). The 2^nd^ most significant gene by RUNNER1 was *PIF1* (*P*= 9.10E-6), which was also the fourth most significant gene detected by RUNNER (*P*= 5.22E-5, see more in Supplementary Table S9a). Pif1 functions as a 5’-3’ DNA helicase present in all eukaryotes. It is an important factor for maintaining mitochondrial DNA, regulating DNA replication and resolving G4-DNA, and might be a promising therapeutic target for age-associated neurodegenerative disorders (48). *DPH1* was another suggestively significant gene by both RUNNER (second rank, *P*= 3.99E-6) and RUNNER1 (fourth rank, *P*= 2.73E-5). *DPH1* encodes an enzyme involved in the biosynthesis of diphthamide, a unique hidtidine residue found only in elongation factor-2 (*EF2*). Eukaryotic EF2 pathway is a major regulator of protein synthesis, synaptic plasticity and memory consolidation and has been implicated in AD pathogenesis (49,50). The estimated coefficients of the predictors of RUNNER in this dataset were similar to that in HSCR (Supplementary Table S6b). The interaction predictor also showed the highest statistical significance.

In contrast, the four alternative methods detected no significant genes. The smallest *P*-values by CMC, KBAC, SKAT and SKATO were 2.87E-4 (*AMOTL2*), 3.0E-4 (*ELN*), 4.57E-4 (*C3orf35*) and 2.4E-4 (*C3orf35*), respectively. None of these genes has been implicated in AD by published studies.

#### Amyotrophic lateral sclerosis (ALS)

ALS is a progressive nervous system disease that affects nerve cells in the brain and spinal cord, which causes loss of muscle control (51). Besides 46 unrelated sporadic ALS patients, this dataset includes eight related ALS patients, all of which were sequenced at exomes (52). We applied RUNNER to evaluate the rare mutation burden at genes among unrelated and all patients, respectively. As shown in Supplementary Figure S8a, even in such a small sample of 46 unrelated patients, the *P*-value QQ plot still followed a uniform distribution *U*[0,1], and the MLFC was only 0.047. The inclusion of 8 related ALS patients from the same family did not significantly disturb the distribution, MLFC = 0.039 (Supplementary Figure S8b), suggesting that RUNNER might also work for samples with partial relatedness, although it is subject to validation in more real datasets. However, RUNNER became slightly inflated when equal weights of 1 were used in this small sample (Supplementary Figure S8c and d). Probably the weight introduced more variation to construct a stable baseline estimation model in RUNNER.

RUNNER detected one significant gene among all the 54 patients, *SOD1* (*P*= 8.70E-9) (see more in Supplementary Table S9b). *SOD1*, encoding an isozyme responsible for destroying free superoxide radicals in the body, is a well-known causal gene of familial ALS (53). All 8 related ALS patients shared a rare missense mutation (c.449T > C, p.I150T) in *SOD1*. Although there were only nine missense mutations in all the patients of this gene, the ultra-rare allele frequencies, deleteriousness functional prediction and gene's lower tolerance to missense or LOF mutation, made RUNNER produce a highly significant *P*-value for mutation burden at this short gene (coding region length is 0.5KB). When eight related samples were removed, the most significant gene detected by RUNNER was *ARHGEF6* (*P*= 5.20E-5, see more in Supplementary Table S9c), which was also the sixth significant gene by RUNNER on all 54 cases. *ARHGEF6*, encoding a guanine nucleotide exchange factor for Rho GTPases. Though there has been no direct reference linking this gene with ALS, aberrations in Rho signaling have been suggested to contribute to the disease of ALS (54).

## DISCUSSION

The present study proposed a novel approach, RUNNER, to evaluate deviation from baseline rare mutation burden across genes in whole-genome or -exome sequencing studies. To our knowledge, this is the first statistical test for genetic mapping based on baseline mutation burden at genes. In contrast, conventional alternative methods were developed based on the deviation of mutation burden between cases and controls. We showed that RUNNER was much more powerful than four widely-used methods based on mutational burden differences between cases and controls (CMC, SKAT, SKATO and KBAC) for genetic association tests at rare variants in simulation studies. Intuitively, the greater power of RUNNER can be attributed to the use of genome-wide mutational burden information to provide more accurate estimates of local mutational burden in the population, especially when the control sample size is small. We also demonstrated that RUNNER was much more robust to population structure than the case-control methods. The enhanced power was reaffirmed by detecting more known or promising candidate susceptibility genes in real data analysis of multiple complex diseases. To facilitate the application of this powerful approach, we have implemented RUNNER as one of the modules on our comprehensive software platform for high throughput sequencing data analysis, KGGSeq (http://pmglab.top/kggseq/).

Besides the burden comparison strategy, the enhanced power of RUNNER is also attributed to multiple methodological innovations. First, the truncated negative binomial distribution fitted mutation counts well. The truncation kept the model-fitting from potential harm by the genes with no or low mutation counts in a small or moderate sample. It also has a data-driven algorithm to determine the appropriate truncation points for the model fitting. Besides, the negative binomial distribution modeled the overdispersion of the mutation counts properly. When the model was replaced with the truncated Poisson distribution, we found that the *P*-values by the regression model became inflated under the null hypothesis (Supplementary Figure S2). Second, RUNNER had a recursive procedure to purify the background gene list. A pure background will lead to a more accurate estimation of the baseline mutation burden. Otherwise, significant genes may introduce an over-estimation of the baseline mutation, which will decrease the deviation and thus reduce the power of RUNNER. Finally, RUNNER has the advantage of optimizing a scale for prior weights at variants to boost the power further. A too large scale may introduce type 1 error inflation, while a too small scale will not enhance the power. RUNNER had an algorithm to automatically explore an optimal scale that minimizes inflation and maximizes the number of significant genes simultaneously. Based on the optimal scale, we showed that the prior weights were able to increase up to 30% or more power in simulation studies while keeping the correct type 1 error rate (Figures 2 and 4). The deviation from the baseline mutation burden has been used for cancer somatic mutations analysis (21). However, it is seldom used for germline rare mutations analysis because of the challenges in modeling the distribution of mutation counts of rare variants. These methodological innovations provide efficient solutions to these challenging issues.

The insensitiveness of RUNNER to population stratification can be explained by its analysis strategy in principle. Instead of comparing allelic frequency between cases and controls as conventional case-control association analyses do, RUNNER essentially calculates the relative difference between the observed rare mutation burden and the estimated baseline rare mutation burden at a gene in patient samples. So, the different ancestry of controls will not affect the calculation (See validations in Figure 3 and Supplementary Figure S3). However, when the cases are from admixed populations with different background mutation rates (say East Asian and African), it would be difficult to select a matched reference population to filter out population private variants and model the gene frequency scores. The use of an unmatched reference population can lead to a significant *P*-value at a gene with the population's private variants (Supplementary Figure S5). This advantage is intriguing for genetic association analysis in rare variants because it is more challenging to correct population stratification in rare variant analysis than in the common variant analysis (15).

RUNNER was originally proposed for unrelated samples. However, in the ALS dataset, we showed that it successfully detected causal genes even when 8 (∼15%) subjects were from the same family, and it did not show systematic inflation of *P*-values (Supplementary Figure S8b). The insensitiveness to subject relatedness is also because both the observed and the estimated baseline mutation burden would increase simultaneously, and their relative difference (i.e. the deviation) was not affected largely. To investigate how the proportion of sample relatedness influences the performance of RUNNER, we expanded the ALS family by mimicking random mating in another simulation experiment based on ALS dataset. As shown in Supplementary Figure S9, when the relatedness in over 30%, multiple genes with private mutations of this family became significant, although there remained no systematic inflation of type 1 error. Therefore, RUNNER should be applied with caution when subjects from a large family dominate a sample.

Besides the systematic simulation studies, the real data application further demonstrated the effectiveness of RUNNER for the genetic mapping of rare variants. In a proof of principle example, RUNNER successfully recaptured (*P*< 2.5E-6) the known causal gene *RET* (38,39), and prioritized another known gene *EDNRB* (39,40) of Hirschsprung disease. These genes were ignored by the four widely-used alternative tests, CMC, SKAT, SKATO and KBAC. In the AD dataset, RUNNER1 showed a significant *P*-value at a very promising candidate susceptibility gene, *NAT1* (*P*= 1.24E-7) (47). In the ALS dataset containing familiar patients, RUNNER successfully detected a causal gene, *SOD1* (*P*= 8.70E-9) (53), in a case-only analysis. However, RUNNER may have decreased power for highly polygenic diseases in which many genes contribute to the phenotype with mild effects. This is because the too many mildly-burdened genes, if not excluded by the recursive procedure, will be considered into the background, elevate the expected mutation counts, and decrease the relative difference between observed and expected mutation counts. In the hypothetical example for intuitive explanation, the *P*-value increased from 2.52E-7 to 0.0021 if the background mutation frequency was biased to be tripled. However, this would only happen when the proportion of mildly-burdened genes with rare mutations is very large. Unfortunately, the proportion is generally unknown. The success of RUNNER in three complex diseases suggests the mildly-burdened genes may not substantially decrease the power for a number of complex diseases.

We used eight predictors under the RUNNER model for the background mutation count of genes in the present study. Although most of the eight predictors were significant and led to the approximately uniform distribution of *P*-values, it did not exclude the usage of other predictors. For example, the sequencing coverage of a gene would also be a potential predictor. However, the sequencing coverage may be data-dependent. When sequencing depth is high enough, almost all genes have enough reads to call reliable genotypes. In this scenario, its impact on the prediction will be small and contribute little to the baseline mutation burden. Besides, it has been noted that the mutation rate tends to be lower in highly expressed genes (46) due to a process termed transcription-coupled repair (55). On the other hand, the mutation rate tends to be higher in regions with late DNA replication timing (56), probably due to the depletion of free nucleotides (57). Studies have suggested that mutation rates may also be associated with epi-genomic features (58). However, these features may be cell-type and developmental stage-dependent. It is often challenging to obtain the right cell type at the right time for a type of disease in practice. Note that the gene frequency score in the model may also be related to the gene expression and replication timing. The former's usage in the regression may partially account for the latter's impact on the mutation counts.

The model of RUNNER is independent of mutation types. Due to abundant data available for validation, we focus on coding and splicing mutations in this paper. It, however, does not preclude its potential extension to rare noncoding mutations. However, additional future work is needed for the extension to noncoding variants. As the evolution selection and genomic features of noncoding variants may differ from coding variants (59), different predictive factors may be needed for the mutation counts in noncoding regions. The biological interpretation of significant results is also more difficult for noncoding genomic regions. A possible way is to analyze sub-noncoding regions separately, e.g. upstream and first intronic regions. Besides, the functional prediction at noncoding variants is less advanced than that at coding variants generally. How the prior weight based on less accurate prediction at noncoding variants influences the power will also be an interesting future work. We performed a preliminary extension of the proposed framework for rare variants in upstream and downstream regions. Although only the gene-frequency score was used, the *P*-values only slightly departed from the uniform distribution (Supplementary Figure S10). This preliminary investigation suggested that the framework RUNNER is extendable for noncoding regions with more effective predictors.

Two limitations should be noted in the present paper. First, the analysis framework does not directly consider covariates to adjust for confounding factors of diseases, e.g. sex and age. For a disease with strong confounding factors, one could exclude patients with a large posterior probability of being a case according to logistic regression with the confounding factors (22). The calculation of posterior probability can be seen in our previous method. However, it should also be noted that RUNNER does not directly detect the association between mutation counts and case-control status. Therefore, it might not be sensitive to the covariates. In a quick investigation, we deliberately misspecified 50 controls as the patients in the Hirschsprung disease dataset to mimic the scenario that genetic factors did not primarily cause 10% of patients. The *P*-values of RUNNER still followed uniform distribution, and the known causal gene *RET* also achieved a significant *P*-value (Supplementary Figure S11 and Supplementary Table S9d). In the AD dataset, RUNNER results for age-adjusted and original phenotypes were also quite similar (Supplementary Table S10). Second, RUNNER will not work in the association analysis only at a few available genes. RUNNER resorts to hundreds of genes to build a baseline model at a time. Therefore, the substantially enhanced power of RUNNER does not mean that it will be a universal replacement of the conventional case-control tests for genetic mapping. However, it may be an effective complementary analysis approach when well-matched control samples are unavailable or case-control analyses are underpowered.

In summary, we proposed a novel and powerful statistical approach, RUNNER, to detect susceptibility genes with rare mutations. Its baseline mutation burden estimation and advanced weighted truncated negative binomial regression may motivate many follow-up methodological studies for genetic mapping from a new angle. As we demonstrated in the applications, the enhanced power of RUNNER may save multiple susceptibility genes missed by widely-used case-control association tests.

## DATA AVAILABILITY

The website of KGGSeq: http://pmglab.top/kggseq/

The source codes of RUNNER and KGGSeq: https://github.com/pmglab/KGGSeq/blob/main/src.zip

The pipeline of RUNNER performed in real datasets of complex diseases analysis: https://github.com/pmglab/KGGSeq/blob/main/RUNNER_pipeline.sh

The pipeline of RUNNER1 performed in real datasets of complex diseases analysis: https://github.com/pmglab/KGGSeq/blob/main/RUNNER1_pipeline.sh

The pipeline of four alternative rare mutation burden tests performed in real datasets of complex diseases analysis: https://github.com/pmglab/KGGSeq/blob/main/RVtests_pipeline.sh

The website of gnomAD: https://gnomad.broadinstitute.org/

The selective expression of genes at REZ: http://pmglab.top/rez/

The source codes of the zero-truncated negative-binomial regression model in the ‘countreg’ R package.

The source codes of the truncated negative-binomial regression model: https://github.com/pmglab/KGGSeq/blob/main/negtrunc.R

The source codes for the semi-simulations: https://github.com/pmglab/VCFSimulator

The tool Picard for removing redundant reads: https://broadinstitute.github.io/picard/

## Supplementary Material

gkab1234_Supplemental_File
