# Supplementary material for: Deviation from baseline mutation burden provides powerful and robust rare-variants association test for complex diseases
Source: Nucleic Acids Res. 2021 Dec 20;50(6):e34. doi: 10.1093/nar/gkab1234 (PMC8989543; doi:10.1093/nar/gkab1234)
Supplement: gkab1234_Supplemental_File [file gkab1234_supplemental_file.pdf]

Deviation from baseline mutation burden provides powerful and robust rare-variants association test for complex diseases

Supplementary Methods

1. Maximum likelihood estimation of coefficients under the truncated negative binomial regression

The probability mass function (PMF) of a negative binomial distribution is

$f(y|\mu_i,\theta) = \frac{\Gamma(y+\theta)}{\Gamma(\theta)\cdot y!}\cdot\frac{\mu_i^y\theta^\theta}{(\mu_i+\theta)^{y+\theta}}$, where $\mu_i = e^{X^T\beta}$, $\theta = e^\gamma$.

And the cumulative distribution function of the distribution is

$\mathrm{F}(\mathrm{X} \le \mathrm{t}|\mu_i;\theta) = \sum_{i=0}^{t}\frac{\Gamma(i+\theta)}{\Gamma(\theta)i!}(\frac{\mu_i}{\mu_i+\theta})^i(\frac{\theta}{\mu_i+\theta})^\theta$.

Given a truncated point t, the PMF a truncated negative binomial distribution is

$g(y|\mu_i,\theta,t) = \frac{f(y|\mu_i,\theta)}{1-F(X\le t|\mu_i,\theta)}, \quad y = t+1, t+2, \cdots$.

So, the likelihood function of the truncated negative binomial distribution of all genes is $\mathrm{L}(\theta,t) = \prod_i\frac{f(\mathrm{y}_i|\mu_i,\theta)}{1-F(X\le t)}$. The log-likelihood function is $\mathrm{lnL}(\theta,t) = \sum_i l_i$, where $l_i = \mathrm{ln}f(\mathrm{y}_i|\mu_i,\theta) - \mathrm{ln}(1-F(X\le t|\mu_i,\theta))$.

We then have the first derivatives of the log-likelihood function:

$$\frac{\partial l_i}{\partial\mu_i} = \frac{\mathrm{y}_i}{\mu_i} - \frac{\mathrm{y}_i+\theta}{\mu_i+\theta} + \sum_{j=0}^{t}\left(\frac{\mathrm{j}}{\mu_i} - \frac{\mathrm{j}+\theta}{\mu_i+\theta}\right)\frac{f(j,\mu_i,\theta)}{1-F(X\le t|\mu_i;\theta)}$$

$$\frac{\partial l_i}{\partial\theta} = \psi(\mathrm{y}_i+\theta) - \psi(\theta) + \ln\frac{\theta}{\mu_i+\theta} + 1 - \frac{\mathrm{y}_i+\theta}{\mu_i+\theta} + \sum_{j=0}^{t}[\psi(\mathrm{j}+\theta) - \psi(\theta) + \ln\frac{\theta}{\mu_i+\theta} + 1 - \frac{\mathrm{j}+\theta}{\mu_i+\theta}]\frac{f(j,\mu_i,\theta)}{1-F(X\le t|\mu_i;\theta)}$$

and $\frac{\partial\mu_i}{\partial\beta} = \mu_i x_i, \frac{\partial\theta}{\partial\gamma} = \theta$, where $\psi(x) = \frac{d(ln\Gamma(x))}{dx} = \frac{\overline{\Gamma(x)}}{\Gamma(x)}$.

Then we can get the derivatives of likelihood function L:

$$\frac{\partial L}{\partial\beta} = \sum_i[\mathrm{y}_i - \mu_i\frac{\mathrm{y}_i+\theta}{\mu_i+\theta} + \sum_{j=0}^{t}\left(j - \mu_i\frac{\mathrm{j}+\theta}{\mu_i+\theta}\right)\frac{\mathrm{f}(j,\mu_i,\theta)}{1-F(X\le t|\mu_i;\theta)}]X$$

$$\frac{\partial L}{\partial \gamma} = \sum_i [\psi(\mathrm{y}_i + \theta) - \psi(\theta) + \ln\frac{\theta}{\mu_i + \theta} + 1 - \frac{\mathrm{y}_i + \theta}{\mu_i + \theta}$$

$$+ \sum_{j=0}^{t} [\psi(\mathrm{j} + \theta) - \psi(\theta) + \ln\frac{\theta}{\mu_i + \theta} + 1$$

$$- \frac{\mathrm{j} + \theta}{\mu_i + \theta}] \frac{f(j, \mu_i, \theta)}{1 - F(X \leq t|\mu_i; \theta)}]\theta$$

Let the above derivatives be equal to zero, and we can obtain the estimated parameters maximizing the log-likelihood. We adopt the optimal function, "optim", in the "stat" R package to calculate the parameters and modify zerotrunc function in the "countreg" R package to expand the original truncated point from zero to a parameter we can select.

2. The deviance residuals of truncated negative binomial regression

We use the deviance residuals to evaluate the deviation of an observed value from the estimated mean by the above truncated negative binomial regression. Here is the definition:

$$e_i = sign(r_i)\sqrt{2|l(y_i|\mu_i^*, \hat{\theta}, t) - l(y_i|\hat{\mu}_i, \hat{\theta}, t)|}$$

Where $l(y_i|\mu, \theta) = \ln[g(y|\mu_i, \theta, t)]$, $r_i = y_i - \hat{y}_i$ and $\hat{y}_i = \frac{\hat{\mu}_i}{1-F(X\leq t|\hat{\mu}_i;\hat{\theta})}$.

The $\hat{\mu}_i$ is the estimated mean given the observed count y_i and estimated $\hat{\theta}$ of a saturated model,

μ_i^* is the root of $\frac{\partial l_i}{\partial \mu_i} = 0$ in interval $[0, y_i]$ which is the root of the formula as below:

$$h(\mu) = y_i - \mu + \sum_{j=0}^{t}(j - \mu)\frac{\mathrm{f}(j|\mu, \hat{\theta})}{1-F(X\leq t|\mu;\hat{\theta})} = 0.$$

Supplementary Figures

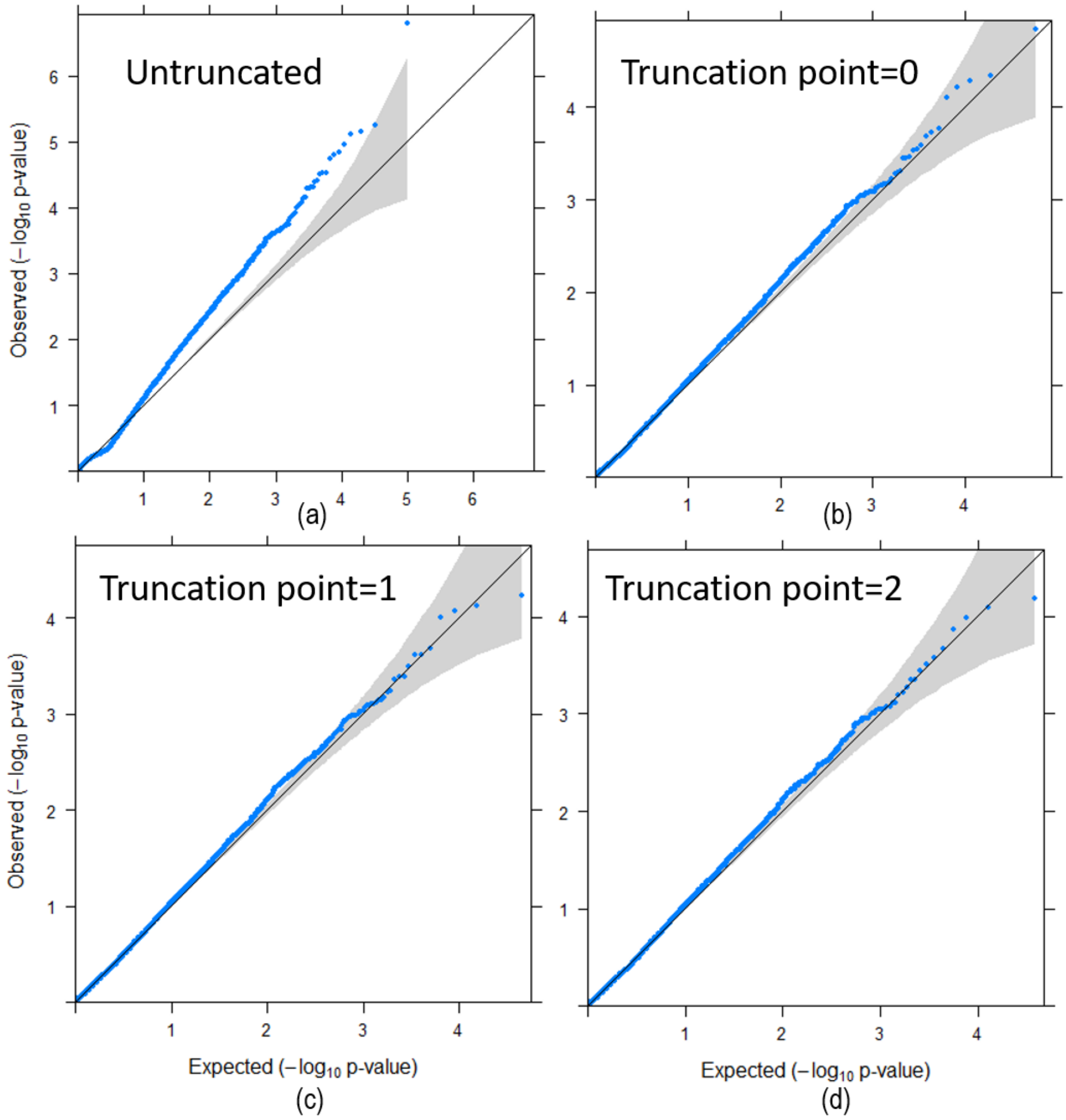

Figure S1. The QQ plots of p-values by simulated data of negative binomial distribution The data were generated by the R scrips: X1 <- runif(n, -1, 1); X2 <- runif(n, -10, 10); Y <- rnbinom(*n*, size = 2.5, mu = exp(0.2 + 0.4×X1+0.8×X2)). In plot (a), the p-values were calculated by the glm.nb() function of the MASS R package. In plots (b-d), the p-values were calculated by our developed truncated negative binomial distribution with different truncation points.

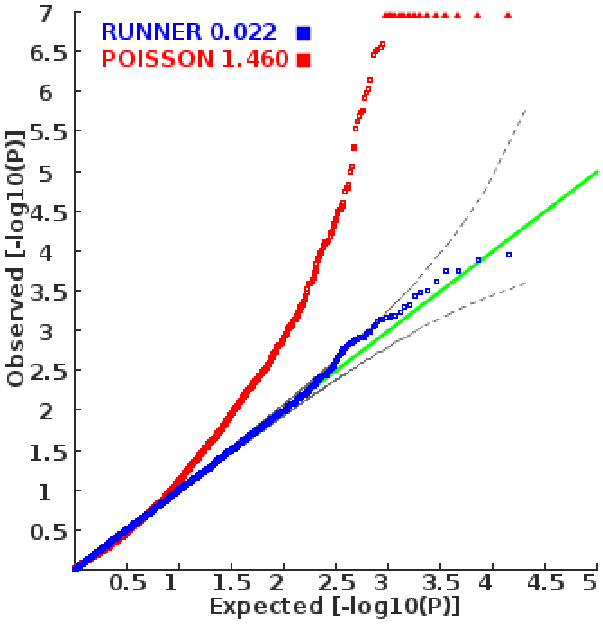

Figure S2. The QQ plot of RUNNER's p-values with different models RUNNER: the proposed truncated negative binomial regression model; POISSON: an alternative truncated Poisson regression model. The dataset included 300 pseudo-cases and 300 healthy controls to investigate type 1 errors under the null hypothesis. In the plot, the values behind the model name denote its corresponding inflation index, MLFC (the mean log fold change). The semi-simulation procedure produced the data based on Hirschsprung’s disease whole-genome sequencing samples.

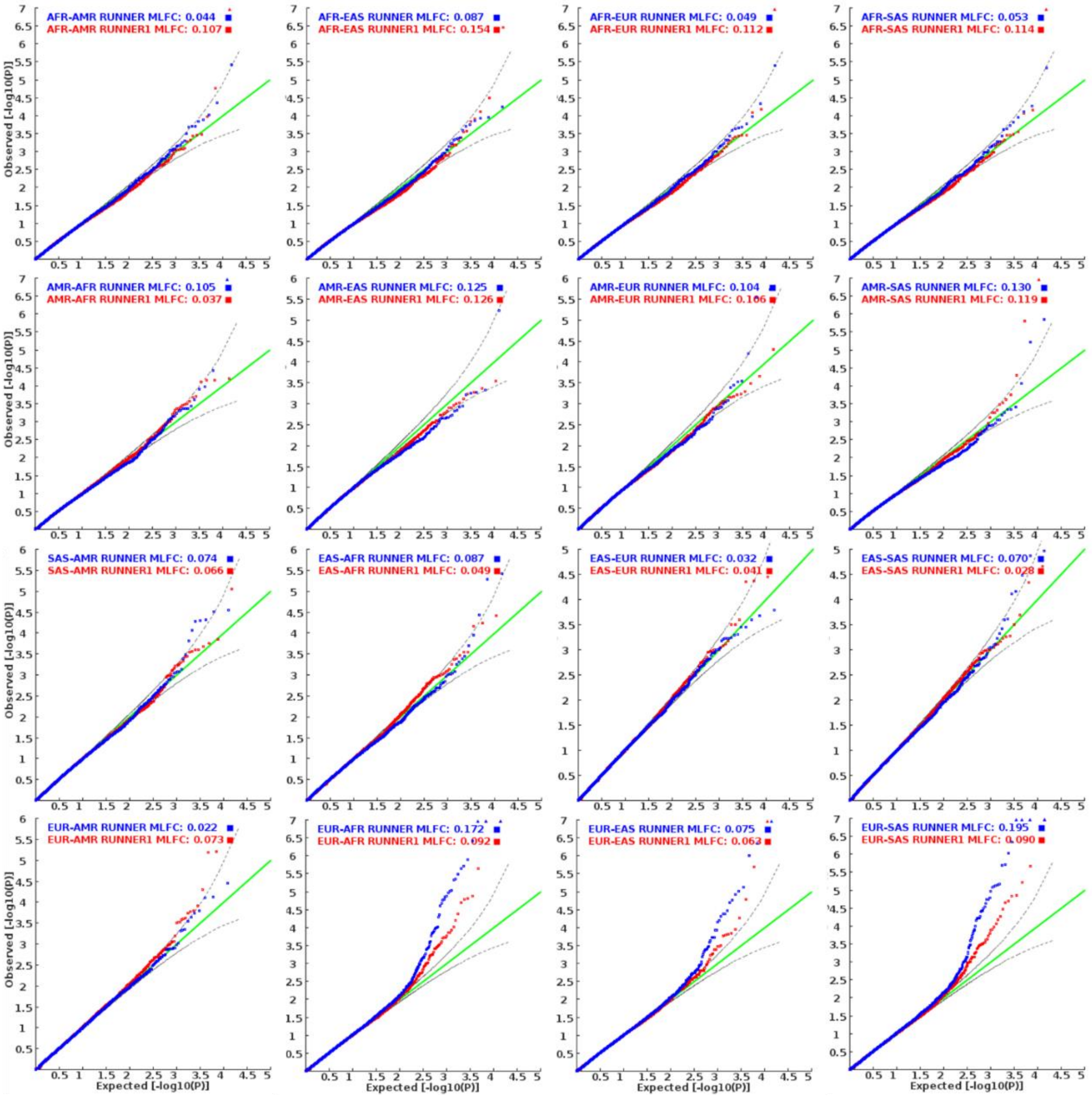

Figure S3. The QQ plots of RUNNER's p-values in 20 samples from stratified populations MLFC: the mean log fold change; RUNNER: the proposed approach (shown as blue points); RUNNER1: the equal-weight version of RUNNER which using original mutation counts at variants (shown as red points). AFR-SAS: the pseudo-patients were from AFR panel while half of the controls were from AFR panel and half of the controls were from SAS panel of the 1000 Genomes Project. All the other 19 population labels denote stratification patterns.

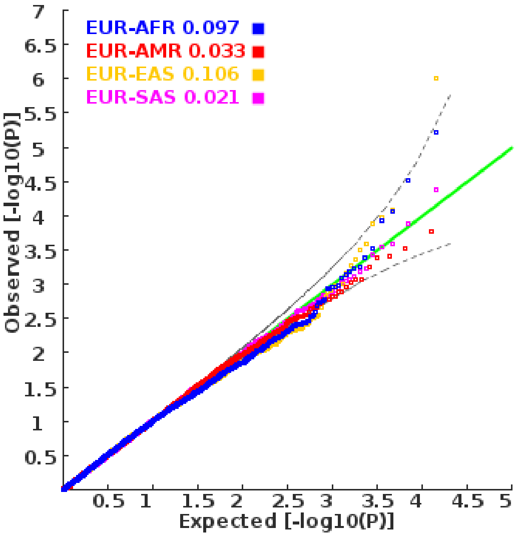

Figure S4. The QQ plots of RUNNER's p-values in four samples from stratified populations The variants with allele frequency >1.5% in pseudo-patients were filtered out. The AFR, AMR, EAS, EUR, and SAS denote African, AdMixed American, East Asian, European and South Asian subjects in the 1000 Genomes Project.

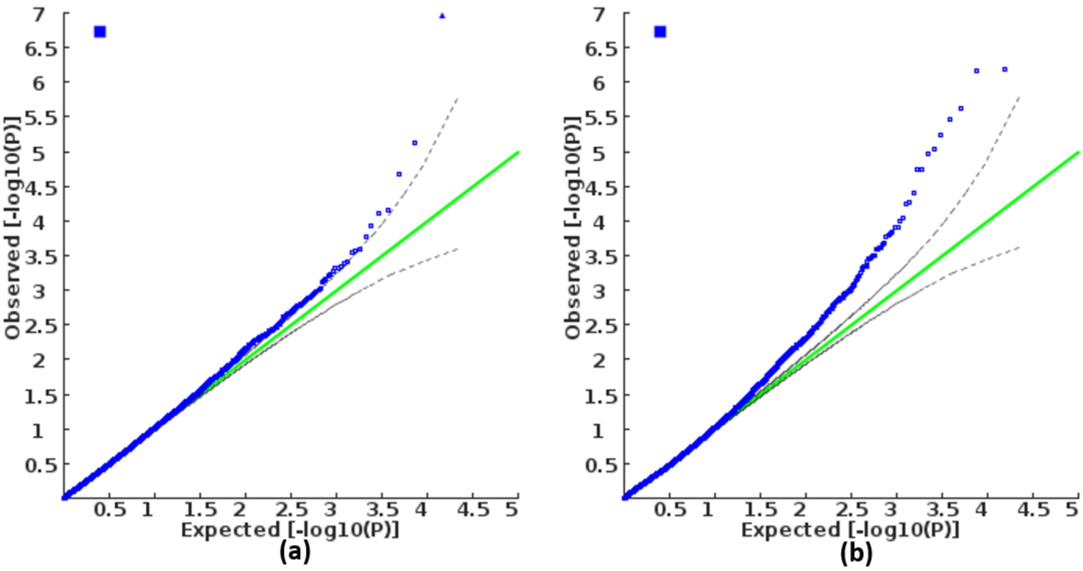

Figure S5. The QQ plots of RUNNER's p-values with unmatched reference a) 503 European pseudo-cases were analyzed based on East Asian reference allele frequencies in the gnomAD database. b) 504 East Asian pseudo-cases were analyzed based on Non-Finnish European reference allele frequencies in the gnomAD database. Variants with MAF >3% were excluded.

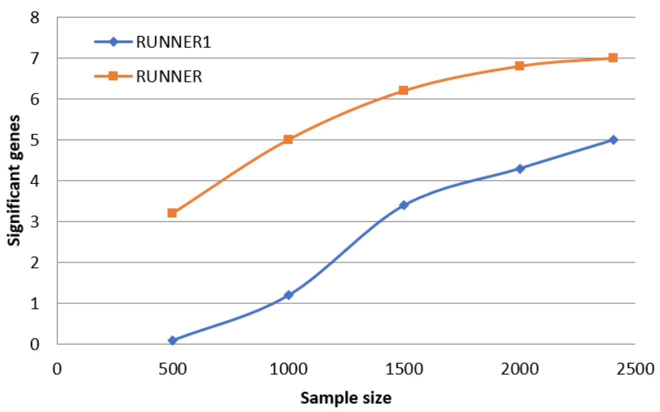

Figure S6. The relationship between the number of significant genes and sample sizes The numbers of significant genes at sample sizes 500, 1000, 1500 and 2000 were averaged from 10 subsamples from 2405 pseudo-cases based on the SG10K Genome Project.

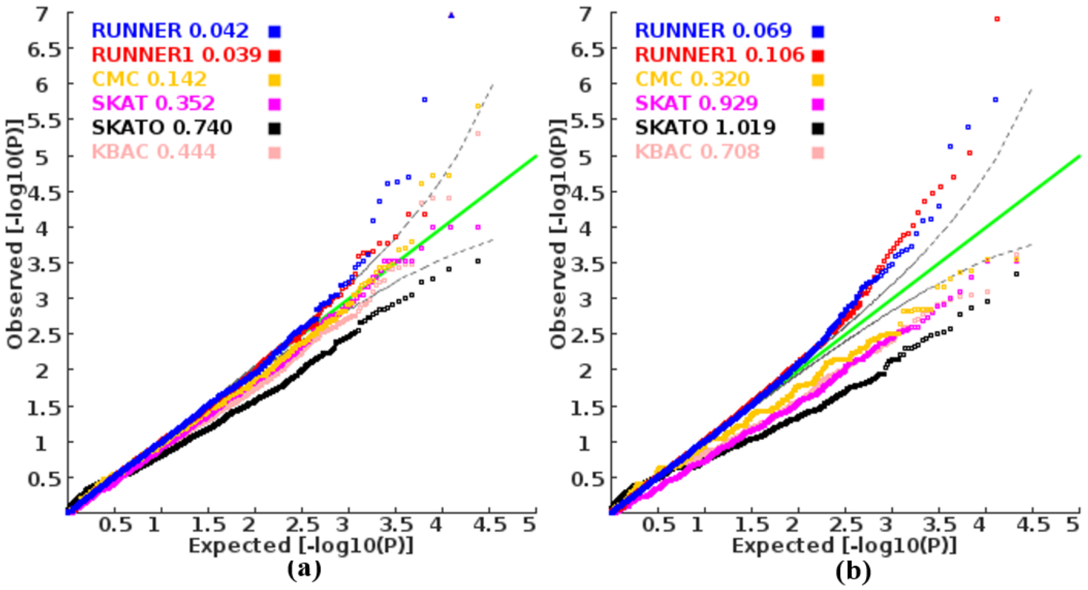

Figure S7. The QQ plots of p-values in real datasets of complex diseases by different methods a) the association p-values at Hirschsprung disease, b) the association p-values at Alzheimer's disease. RUNNER, the proposed method; RUNNER1, the equal weight version of RUNNER using original mutation counts at variants; CMC, combined multivariate and collapsing method; KBAC, the sequence kernel association test; SKAT, the kernel-based adaptive clustering method; SKATO, the optimized-SKAT method. The values behind the model name denote its corresponding inflation index, MLFC (the mean log fold change), in the plots.

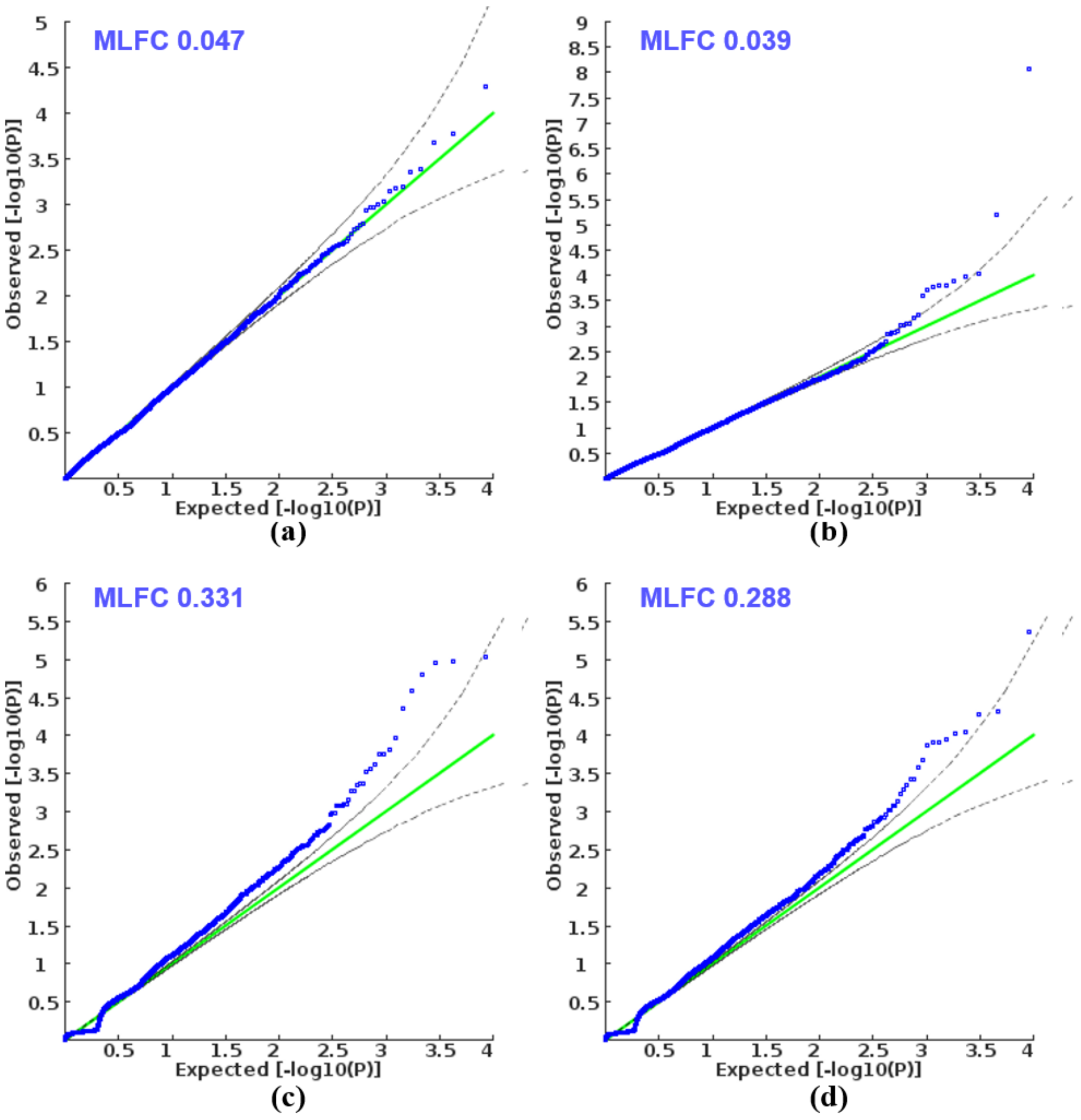

Figure S8. The QQ plots of RUNNER's p-values in amyotrophic lateral sclerosis dataset a) RUNNER in 46 sporadic patients only, b) RUNNER in the 46 sporadic and 8 related patients from the same family, c) RUNNER1 in 46 sporadic patients only, d) RUNNER1 in the 46 sporadic and 8 related patients from the same family. RUNNER, the proposed method; RUNNER1, the equal-weight version of RUNNER, which uses original mutation counts at variants. MLFC: the mean log fold change.

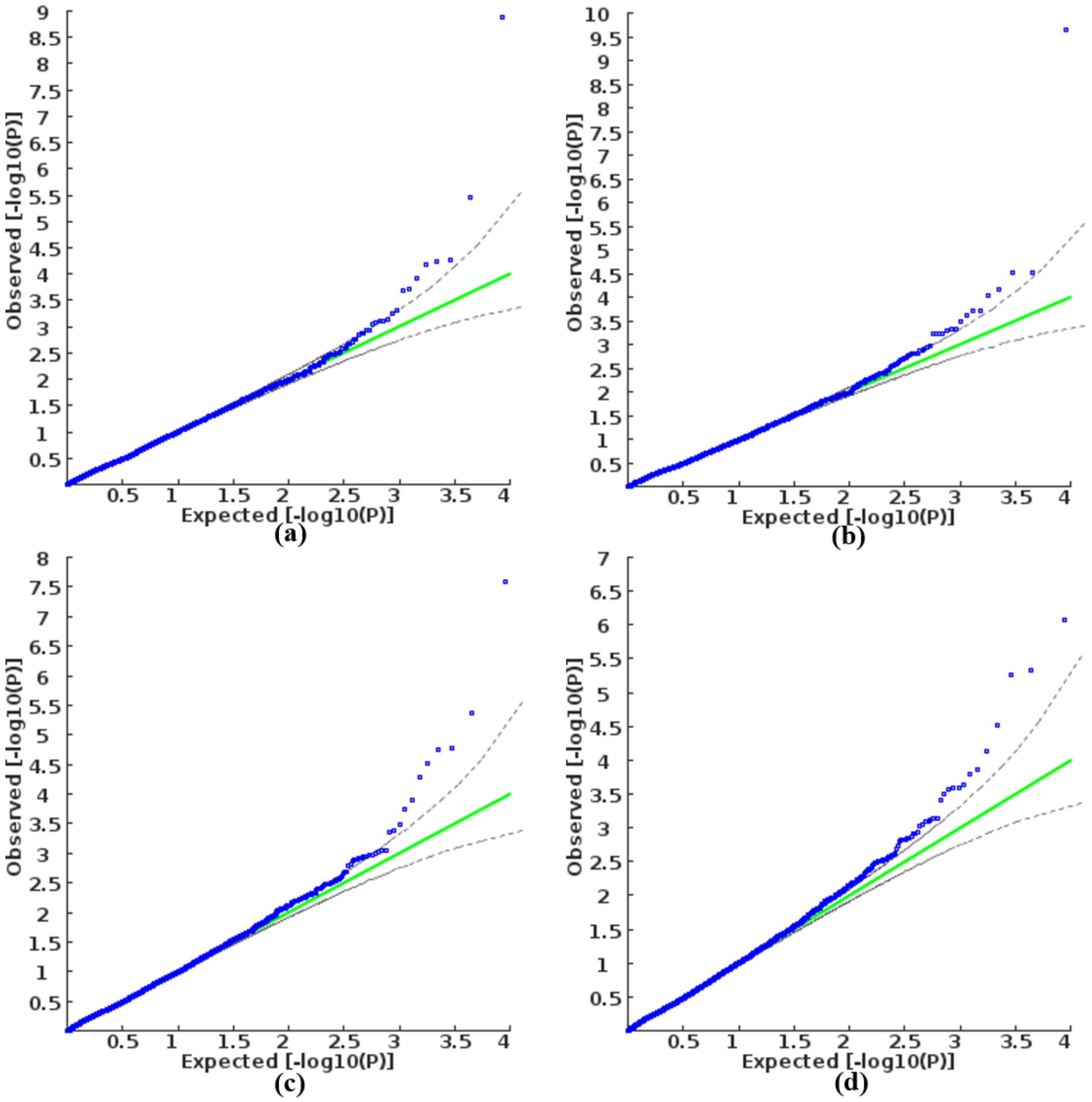

Figure S9. The QQ plots of RUNNER's p-values in ALS disease dataset with different proportions of related samples a) 15% subjects were related and from the sample pedigree; b) 22% subjects were related and from the sample pedigree; c) 30% subjects were related and from the sample pedigree; d) 37% subjects were related and from the sample pedigree.

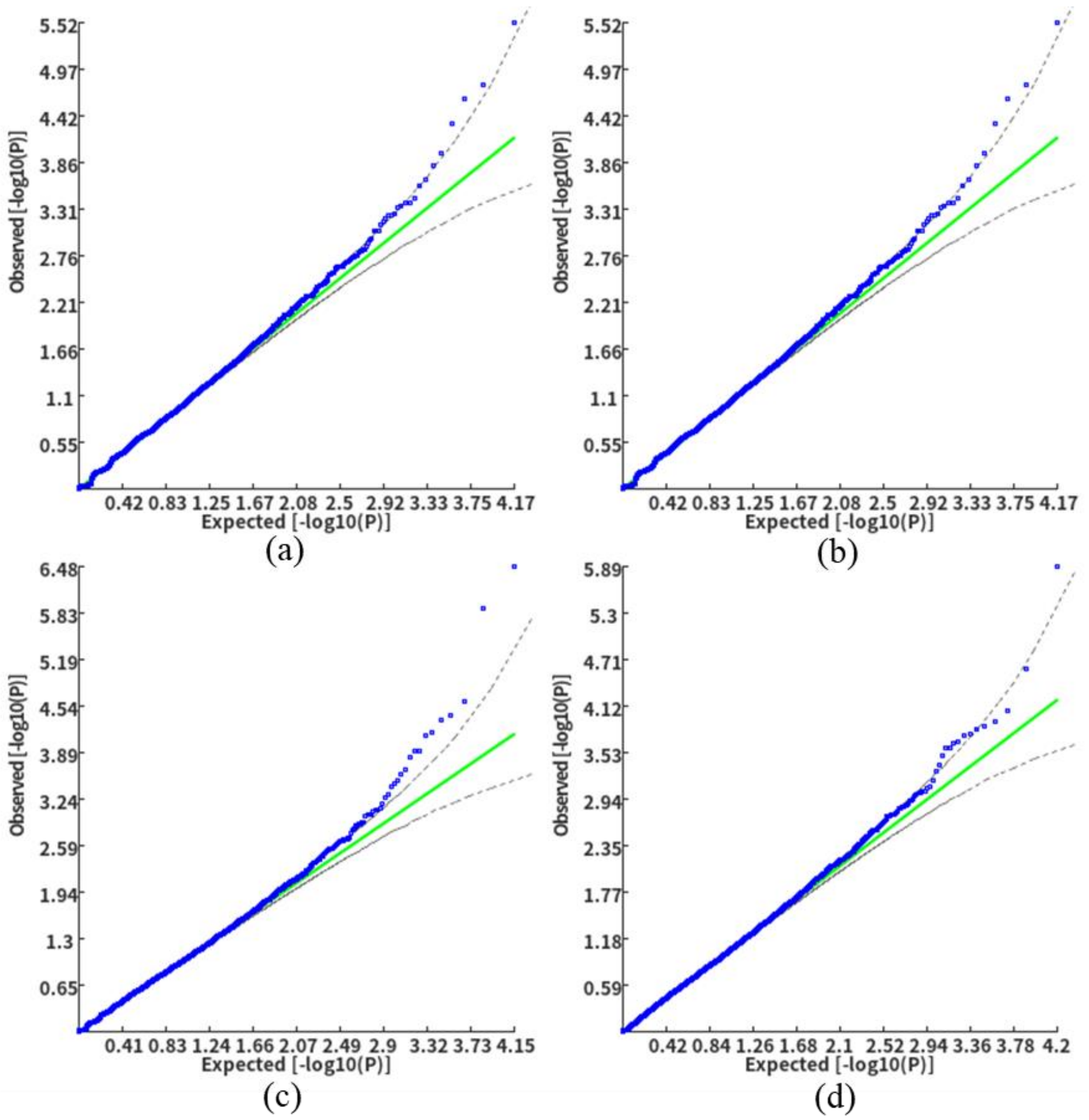

Figure S10. The QQ plots of RUNNER for upstream and downstream variants Only the gene-frequency score was used as a predictor in this RUNNER model. a) RUNNER1 in 100 cases and 100 controls; b) RUNNER in 100 cases and 100 controls; c) RUNNER1 in 200 cases and 200 controls; d) RUNNER in 200 cases and 200 controls. RUNNER, the proposed method; RUNNER1, the equal-weight version of RUNNER which uses original mutation counts at variants. The samples were randomly drawn from the Hirschsprung's disease whole-genome sequencing dataset.

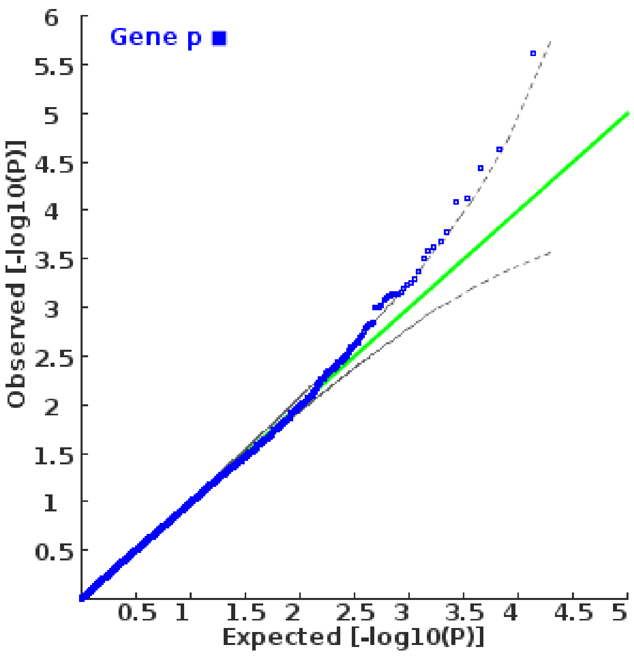

Figure S11. The QQ plot of RUNNER's p-values in the Hirschsprung disease dataset with 10% wrongly specified patient samples

Supplementary Tables

Table S1. The expected runtime of RUNNER with different sample sizes

Size	RUNNER(Min.)	RUNNER1(Min.)
**75**	9.5	4.1
**150**	11.0	4.4
**300**	11.9	4.6
**450**	13.2	4.7

Note: The sample used for the testing was the Hirschsprung disease sample; the hardware includes Intel(R) Core(TM) i9-9900 CPU @ 3.10GHz and DDR4 2666 MT/s RMA. Twelve CPU threads were created. The time in each cell refers to the runtime for the whole procedure, including loading and parsing genotypes, gene feature annotation, functional prediction, parameter estimation, and grid searching. RUNNER is the proposed approach; RUNNER1 is the equal weight (weight=1) version of RUNNER, which uses original mutation counts at variants.

Tables S2. Prediction scores combined to produce functional weights

ID	Approach	Description
**1**	SIFT_score	An algorithm predicts whether a single amino acid substitution affects protein function or not. It assumes that important amino acids in a protein sequence should be conserved throughout evolution and substitutions at highly conserved sites are expected to affect protein function. A small score indicates a high chance for a substitution to damage the protein function. [PMID: 12824425]
**2**	Polyphen2_HDIV_score	Polyphen2_HDIV_score based on HumDiv, i.e. hdiv_prob. The score ranges from 0 to 1, based on HumDiv. [PMID: 12202775]
**3**	Polyphen2_HVAR_score	Polyphen2_HVAR_score ranges from 0 to 1, generated by the HumVar (instead ofHumDiv) trained model as it is preferred for the diagnosis of Mendelian diseases, which predicts the possible impact of an amino acid substitution on the structure and function of a human protein using straightforward physical and comparative considerations by an iterative greedy algorithm. A variant with larger score has a higher possibility to damage the protein function. [PMID: 12202775]
**4**	LRT_score	LRT score, based on a comparative genomics data set of 32 vertebrate species, ranges from 0 to 1. A larger score indicates a larger deleterious effect. It employed a likelihood ratio test to assess variant deleteriousness. The identified deleterious variants, which are likely to be unconditionally deleterious, could disrupt highly conserved amino acids within protein-coding sequences. [PMID: 19602639]
**5**	MutationTaster_score	MutationTaster assesses the affection of the disease-causing potential of a sequence variant by a naive Bayes classifier employing multiple resources such as evolutionary conservation, splice-site changes, loss of protein features and changes that might affect mRNA level. The scores are in [0, 1]. The larger scores are more likely to cause a human disease. [PMID: 20676075]

**6**	MutationAssessor_score	MutationAssessor is used to estimate the functional impact of a variant, giving predicted functional (high, medium) and predicted non-functional (low, neutral). [PMID: 21727090]
**7**	FATHMM_score	FATHMM default score is weighted for human inherited-disease mutations with Disease Ontology. When it is below -1.5, the corresponding NS is predicted as "D(AMAGING)"; otherwise it is predicted as "T(OLERATED)". If there are multiple scores associated with the same NS due to isoforms, the smallest score (most damaging) should be considered. [PMID: 23033316]
**8**	VEST3_score	VEST 3.0 score. Score ranges from 0 to 1. The larger the score the more likely the mutation may cause functional change. In case there are multiple scores for the same variant, the largest score (most damaging) is presented. [PMID: 23819870]
**9**	PROVEAN_score	PROVEAN score (PROVEANori) changes from -14 to 14. The SNP with smaller score has more damaging effect. [PMID: 25851949]
**10**	CADD_raw	Combined Annotation Dependent Depletion (CADD) score is used to predict the functional impact of a SNP. The SNP with larger score likely has more damaging effect. The word "Raw" means the ADD scores come straight from the CADD model. [PMID: 24487276]
**11**	M-CAP_score	M-CAP score is a pathogenicity likelihood score, short for the Mendelian Clinically Applicable Pathogenicity. M-CAP is a clinical pathogenicity classifier which eliminates a majority of variants of uncertain significance in clinical exomes at high sensitivity. [PMID: 27776117]
**12**	MutPred_score	MutPred is a computational model, which can predict whether the mutation is associated with the specific molecular mechanism resulting in the disease states. It involves three attributes for classification, based on predicted protein structure and dynamics, functional properties and (amino acid sequence and evolutionary information respectively. [PMID:19734154]
**13**	DANN_score	DANN is a deep learning approach for annotating the pathogenicity of genetic mutations. It trains a deep neural network (DNN) with the same feature set and training data as CADD. DNNs can capture nonlinear relationships among features and are better suited than SVMs for problems with a large number of samples and features. [PMID: 25338716]
**14**	fathmm-MKL_coding_score	fathmm-MKL_coding_score is generated from MKL classifier to predict the functional consequences of both coding and non-coding sequence substitutions from various genomic annotations and weights the significance of each component annotation source.
**15**	Eigen-raw	Eigen is an unsupervised approach to integrate different functional genomic annotations for coding and noncoding variants. For non-synonymous coding variants, it involves three different blocks one block with protein function scores, a second block with evolutionary conservation annotations, and a third block with allele frequencies respectively. For noncoding and synonymous coding variants, it includes one block with evolutionary conservation annotations, a second block

		with regulatory annotations, and a third block with allele frequencies. [PMID: 26727659]
**16**	GenoCanyon_score	GenoCanyon is an unsupervised statistical learning annotation method that is used toIdentify functional regions in the human genome.It is able to predict many of the known functional regions, as a unique and powerful tool for whole-genome annotation. [PMID:26015273]
**17**	integrated_fitCons_score	The fitness consequence (fitCons) scores measures the potential genomic function based on evolution. The integrated_fitCons_score integrates the fitCons scores generated from three human cell types: human umbilical vein epithelial cells (HUVEC), H1 human embryonic stem cells (H1 hESC), and lymphoblastoid cells (GM12878). [PMID: 25599402]
**18**	GERP++_RS	RS score, the larger the score indicates the more conserved the site. Two parts constitute the GERP++ algorithm. One component is calculation of position-specific constraint scores for each column of a multiple alignment; the other is subsequent aggregation of neighboring columns into segments that score significantly higher than expected by chance. [PMID: 21152010]
**19**	SiPhy_29way_logOdds	SiPhy score ranges from 0 to 37.9718 in dbNSFP, based on 29 mammals' genomes. The larger the score suggests the more conserved the site. [PMID: 19478016]

Table S3. Variants assumed as susceptibility mutations in simulation experiments (based on Hirschsprung sample)

Gene	Chrom	Position	ReferenceAllele	MutantAllele	Assumed Frequency in Patients	Ensemble Prediction Score by KGGSeq
TIE1	1	43770594	T	A	0.0075	0.3068
TIE1	1	43770600	G	T	0. 0075	0.2599
TIE1	1	43770765	G	A	0. 0075	0.4427
TIE1	1	43770807	G	C	0. 0075	0.226
TCF4	18	52895475	T	C	0.005	0.0087
TCF4	18	52895494	C	T	0. 005	0.26
TCF4	18	52901788	G	T	0. 005	0.5984
TCF4	18	52895552	C	A	0. 005	0.5082
TCF4	18	52895555	T	G	0. 005	0.1991

Table S4. Variants assumed as susceptibility mutations in simulation experiments (based on the SG10K Genome Project)

Gene	Chrom	Position	ReferenceAllele	MutantAllele	Assumed Frequency in Patients	Ensemble Prediction Score by KGGSeq
CX3CR1	3	39307621	C	G	0.005	0.561724
CX3CR1	3	39307649	A	C	0.005	0.328656
CX3CR1	3	39307738	G	A	0.005	0.017947
AIG1	6	143458087	G	A	0.005	0.603612
AIG1	6	143654466	T	G	0.005	0.616273

AIG1	6	143458087	G	A	0.005	0.603612
PRR5L	11	36453436	G	A	0.005	0.377984
PRR5L	11	36472767	C	G	0.005	0.495116
PRR5L	11	36472802	T	C	0.005	0.630623
SLX1B	16	30206213	T	A	0.005	0.507737
SLX1B	16	30206339	C	T	0.005	0.302535
SLX1B	16	30208409	G	A	0.005	0.27306
UBBP4	17	21731086	C	T	0.005	0.106965
UBBP4	17	21731541	C	T	0.005	0.126477
UBBP4	17	21731581	T	C	0.005	0.325996
RDH8	19	10124207	G	A	0.005	0.987109
RDH8	19	10129416	C	A	0.005	0.946275
RDH8	19	10129478	G	C	0.005	0.292309
ELOF1	19	11664828	G	A	0.005	0.161261
ELOF1	19	11664877	C	T	0.005	0.680536
ELOF1	19	11664885	C	T	0.005	0.108549
RDH13	19	55556532	A	T	0.005	0.165227
RDH13	19	55556585	C	T	0.005	0.58193
RDH13	19	55559746	G	T	0.005	0.589632
BTBD3	20	11900478	T	C	0.005	0.427475
BTBD3	20	11903872	A	C	0.005	0.45828
BTBD3	20	11903986	G	A	0.005	0.58144
EBPL	13	50237193	C	T	0.005	0.368064
EBPL	13	50243981	T	G	0.005	0.440635
EBPL	13	50243967	A	T	0.005	0.436624

Table S5. Real high-throughput sequencing datasets used in the present paper

Name	Sequencing platform	Number of Patients	Number of Controls	Initial Variants after QC	Rare Non-synonymous Variants	Original Publications
**Hirschsprung disease (HSCR)**	Illumina HiSeq X Ten (WGS)	443	493	334840 (including 9998 Indels, coding regions only)	216704 (including 9893 Indels)	PMID: 30217742
**Alzheimer's disease (AD)**	Illumina HiSeq 2000 (WES)	246	172	411442 (including 5008 Indels)	99539 (including 4406 Indels)	PMID: 29656768
**Amyotrophic lateral sclerosis (ALS)**	Illumina HiSeq 2000 (WES)	54 (contain 8 related)	0	205943 (including 2095 Indels)	30033 (including 1538 Indels)	PMID: 28709720
		46	0	205467 (including 2092 Indels)	29947 (including 1523 Indels)	

Notes: Rare Non-synonymous Variants include missense, start-loss, stop-loss, stop-gain, splicing, frameshift and non-frameshift variants with minor allele frequency$\leqslant$1% in reference populations (East Asian panel of the gnomAD). Indels include frameshift and non-frameshift

variants. Abbreviations: WGS, whole-genome sequencing; WES, whole-exome sequencing; QC, quality control.

Table S6. The estimated parameters of 8 predictors by RUNNER in various real datasets of complex diseases

(a) Hirschsprung disease dataset

Variable	Estimate	Std. Error	z value	Pr(>|z|)
**RegionLength**	0.0693	0.0089	7.7622	8.35E-15
**EAS**	11.8344	0.4004	29.5593	4.99E-192
**RegionLength_EAS**	-0.2115	0.0039	-53.6085	0
**mu_mis**	0.0061	0.0010	6.2438	4.27E-10
**mu_lof**	0.0734	0.0092	7.9393	2.03E-15
**oe_mis**	0.5431	0.0388	14.0136	1.29E-44
**oe_lof**	0.0838	0.0195	4.3027	1.69E-05
**ExonGC**	0.4679	0.0829	5.6416	1.68E-08

(b) Alzheimer's disease dataset

Variable	Estimate	Std. Error	z value	Pr(>|z|)
**RegionLength**	0.0220	0.0112	1.9704	4.88E-02
**EAS**	6.4959	0.2073	31.3403	1.32E-215
**RegionLength_EAS**	-0.0871	0.0020	-42.6165	0
**mu_mis**	0.0043	0.0012	3.6361	2.77E-04
**mu_lof**	0.0957	0.0107	8.9698	2.97E-19
**oe_mis**	0.4971	0.0429	11.5785	5.30E-31
**oe_lof**	0.0082	0.0221	0.3710	7.11E-01
**ExonGC**	0.1527	0.0952	1.6045	1.09E-01

(c) 46 sporadic and 8 related Amyotrophic lateral sclerosis patients

Variable	Estimate	Std. Error	z value	Pr(>|z|)
**RegionLength**	0.0085	0.0107	0.7958	4.26E-01
**EAS**	2.4101	0.1001	24.0739	4.70E-128
**RegionLength_EAS**	-0.0266	0.0011	-24.0123	2.07E-127
**mu_mis**	0.0012	0.0013	0.9546	3.40E-01
**mu_lof**	0.0740	0.0124	5.9886	2.12E-09
**oe_mis**	0.1726	0.0539	3.2056	1.35E-03
**oe_lof**	0.0074	0.0286	0.2586	7.96E-01
**ExonGC**	-0.1868	0.1216	-1.5362	1.24E-01

(d) 46 sporadic Amyotrophic lateral sclerosis patients

Variable	Estimate	Std. Error	z value	Pr(>|z|)
**RegionLength**	0.0035	0.0105	0.3331	7.39E-01
**EAS**	2.1713	0.0984	22.0595	7.74E-108
**RegionLength_EAS**	-0.0235	0.0011	-21.3887	1.70E-101
**mu_mis**	0.0013	0.0012	1.0437	2.97E-01
**mu_lof**	0.0711	0.0122	5.8346	5.39E-09

**oe_mis**	0.1682	0.0537	3.1342	1.72E-03
**oe_lof**	-0.0043	0.0288	-0.1477	8.83E-01
**ExonGC**	-0.2424	0.1233	-1.9663	4.93E-02

Notes: RegionLength and ExonGC are the length (kb) and GC content in exon regions of the gene. EAS refers to the gene frequency scores calculated from the East Asian reference population (EAS) in gnomAD. RegionLength_EAS is the interaction factor generated by multiplying RegionLength and gene frequency scores (allele frequency of EAS was adopted in this analysis). mu_mis and mu_lof refer to the gene's mutation rate based on missense variants and loss-of-function variants in its canonical transcript, respectively. oe_mis and oe_lof refer to the gene's observed over expected ratio at missense and loss-of-function variants obtained from gnomAD, respectively.

Table S7. The rare non-synonymous mutations of *RET* used by RUNNER in Hirschsprung disease dataset

Chr	Start Position Hg19	Reference / Alternative Allele	Most Important Gene Feature	Reference Homozygous Genotype Number in Patients	Heterozygous Genotype Number in Patients	Alternative Homozygous Genotype Number in Patients	Alternative allele frequencies in patients	Reference Homozygous Genotype Number in controls	Heterozygous Genotype Number in controls	Alternative Homozygous Genotype Number in controls	Alternative allele frequencies in controls	Alternative allele frequencies in EAS
**10**	43595990	G/A	missense	442	1	0	0.0011	493	0	0	0.0000	0.0000
**10**	43596026	A/G	missense	442	1	0	0.0011	493	0	0	0.0000	0.0000
**10**	43596128	C/T	missense	442	1	0	0.0011	493	0	0	0.0000	0.0000
**10**	43597792	C/T	missense	442	1	0	0.0011	493	0	0	0.0000	0.0000
**10**	43597793	G/A	missense	413	29	1	0.0350	481	11	0	0.0112	0.0098
**10**	43597849	C/T	missense	442	1	0	0.0011	493	0	0	0.0000	0.0000
**10**	43597916	C/T	missense	442	1	0	0.0011	493	0	0	0.0000	0.0000
**10**	43597976	G/C	missense	442	1	0	0.0011	492	0	0	0.0000	0.0000
**10**	43600606	A/G	missense	439	4	0	0.0045	492	0	0	0.0000	0.0010
**10**	43601905	A/C	missense	442	1	0	0.0011	493	0	0	0.0000	0.0000
**10**	43601945	G/A	missense	442	1	0	0.0011	493	0	0	0.0000	0.0000
**10**	43601972	C/T	missense	442	1	0	0.0011	493	0	0	0.0000	0.0000
**10**	43601986	G/A	missense	442	1	0	0.0011	493	0	0	0.0000	0.0000
**10**	43601990	G/T	missense	442	1	0	0.0011	493	0	0	0.0000	0.0000
**10**	43601995	G/A	missense	442	1	0	0.0011	493	0	0	0.0000	0.0001
**10**	43604598	G/A	missense	442	1	0	0.0011	493	0	0	0.0000	0.0000
**10**	43606776	C/A	stopgain	442	1	0	0.0011	492	0	0	0.0000	0.0000
**10**	43606850	G/A	missense	442	1	0	0.0011	493	0	0	0.0000	0.0000
**10**	43609001	CAG/C--	frameshift	442	1	0	0.0011	493	0	0	0.0000	0.0000
**10**	43609027	G/C	missense	442	1	0	0.0011	493	0	0	0.0000	0.0000
**10**	43609070	G/C	missense	442	1	0	0.0011	493	0	0	0.0000	0.0000
**10**	43609942	G/A	missense	442	1	0	0.0011	493	0	0	0.0000	0.0000
**10**	43613884	A/G	missense	440	3	0	0.0034	493	0	0	0.0000	0.0000
**10**	43615652	G/A	splicing	442	1	0	0.0011	493	0	0	0.0000	0.0000

**10**	43622131	C/T	stopgain	442	1	0	0.0011	493	0	0	0.0000	0.0000
**10**	43622176	AT/A-	frameshift	442	1	0	0.0011	493	0	0	0.0000	0.0000

Table S8. The rare non-synonymous mutations of *NAT1* used by RUNNER in Alzheimer's disease dataset

Chr	Start Position Hg19	Reference / Alternative Allele	Most Important Gene Feature	Reference Homozygous Genotype Number in Patients	Heterozygous Genotype Number in Patients	Alternative Homozygous Genotype Number in Patients	Alternative allele frequencies in patients	Reference Homozygous Genotype Number in controls	Heterozygous Genotype Number in controls	Alternative Homozygous Genotype Number in controls	Alternative allele frequencies in controls	Alternative allele frequencies in EAS
**8**	18080001	G/A	missense	233	13	0	0.0264	169	2	0	0.0058	0.0072
**8**	18080196	T/G	missense	232	14	0	0.0285	169	2	0	0.0058	0.0076

Table S9. Top 10 genes prioritized by RUNNER in various real datasets of complex diseases

(a) Alzheimer's disease dataset

Gene Symbol	Minor Alleles	Missense Alleles	LOF Alleles	Region Length	mu_mis	mu_lof	oe_mis	oe_lof	ExonGC	Residual	P	$Residual_1$	P_1
NAT1	27	27	0	1.074	8.540	0.697	1.106	1.399	0.403	2.179	1.47E-02	5.159	1.24E-07
PIF1	24	23	1	2.249	18.541	1.407	0.950	0.948	0.612	3.880	5.22E-05	4.286	9.10E-06
CXCL16	18	18	0	0.847	7.517	0.454	0.921	0.747	0.577	3.259	5.59E-04	4.104	2.03E-05
DPH1	21	18	3	1.392	15.838	1.328	1.030	0.983	0.597	4.466	3.99E-06	4.035	2.73E-05
PEX3	14	14	0	1.182	8.904	1.526	0.759	0.309	0.353	2.769	2.81E-03	3.989	3.32E-05
NIPAL1	16	16	0	1.263	9.214	0.651	0.867	0.976	0.484	4.650	1.66E-06	3.922	4.39E-05
CRK	11	11	0	1.208	9.618	0.963	0.504	0.069	0.472	2.342	9.59E-03	3.836	6.24E-05
ZNF814	25	6	19	3.400	20.812	0.132	1.125	0.650	0.497	3.132	8.69E-04	3.733	9.46E-05
ZNF479	22	9	13	1.595	11.867	0.302	1.204	1.015	0.398	1.571	5.81E-02	3.661	1.26E-04
IL10RA	19	19	0	1.772	17.630	1.022	0.900	0.347	0.564	2.376	8.76E-03	3.655	1.29E-04

(b) 46 sporadic and 8 related Amyotrophic lateral sclerosis patients

Gene Symbol	Minor Alleles	Missense Alleles	LOF Alleles	Region Length	mu_mis	mu_lof	oe_mis	oe_lof	ExonGC	Residual$_1$	P_1	Residual	P
SOD1	9	9	0	0.490	4.439	0.274	0.747	0.311	0.487	4.446	4.37E-06	5.636	8.70E-09
ABCD1	8	8	0	2.446	32.839	1.338	0.719	0.000	0.637	3.471	2.59E-04	4.370	6.22E-06
HPCAL4	5	5	0	0.591	6.999	0.322	0.754	0.935	0.500	2.703	3.44E-03	3.737	9.30E-05
EFHC2	9	9	0	2.325	18.596	1.406	0.888	0.000	0.376	3.527	2.10E-04	3.704	1.06E-04
POLR1E	6	6	0	1.681	12.679	1.423	0.864	0.587	0.498	2.667	3.83E-03	3.650	1.31E-04
ARHGEF6	7	7	0	2.441	20.517	2.586	0.774	0.029	0.434	3.028	1.23E-03	3.606	1.55E-04
FAHD2A	6	6	0	0.980	9.949	0.829	0.932	0.725	0.549	2.696	3.50E-03	3.602	1.58E-04
NMRAL1	9	9	0	1.020	12.022	0.656	1.151	0.762	0.590	3.368	3.79E-04	3.582	1.70E-04
TFF2	6	6	0	0.410	4.792	0.378	0.854	1.302	0.538	2.959	1.54E-03	3.548	1.94E-04
PPM1J	8	7	1	1.568	15.831	1.485	0.889	0.700	0.633	3.101	9.65E-04	3.488	2.44E-04

(c) 46 sporadic Amyotrophic lateral sclerosis patients

Gene Symbol	Minor Alleles	Missense Alleles	LOF Alleles	Region Length	mu_mis	mu_lof	oe_mis	oe_lof	ExonGC	Residual$_1$	P_1	Residual	P
ARHGEF6	7	7	0	2.441	20.517	2.586	0.774	0.029	0.434	3.337	4.23E-04	3.88	5.20E-05
NMRAL1	7	7	0	1.020	12.022	0.656	1.151	0.762	0.590	3.072	1.06E-03	3.59	1.68E-04
PPM1J	7	7	0	1.568	15.831	1.485	0.889	0.700	0.633	3.141	8.42E-04	3.53	2.08E-04
A4GALT	9	4	4	1.067	17.604	0.372	1.035	0.946	0.605	4.049	2.58E-05	3.35	4.08E-04
COQ3	7	7	0	1.145	8.756	0.648	1.015	0.846	0.434	3.326	4.41E-04	3.32	4.45E-04
CXCR1	5	3	0	1.058	12.241	0.405	1.082	1.238	0.526	2.330	9.91E-03	3.22	6.48E-04
C10orf53	5	5	0	0.624	3.573	0.115	0.856	0.000	0.495	2.898	1.88E-03	3.21	6.69E-04
RP2	5	5	0	1.078	9.866	0.692	0.845	0.000	0.355	2.755	2.94E-03	3.19	7.22E-04
C1orf127	14	12	2	2.532	26.024	1.597	0.914	0.723	0.603	4.250	1.07E-05	3.11	9.37E-04
BSND	6	6	0	0.983	11.192	0.506	0.987	1.190	0.560	2.843	2.23E-03	3.08	1.02E-03

(d) Hirschsprung disease dataset with 10% wrongly specified patient samples

Gene Symbol	Minor Alleles	Missense Alleles	LOF Alleles	Region Length	mu_mis	mu_lof	oe_mis	oe_lof	ExonGC	Residual	P
RET	65	60	5	3.477	44.853	2.678	0.896	0.039	0.585	4.575	2.39E-06
LRRC36	44	36	6	2.363	19.052	1.815	0.835	0.658	0.454	4.073	2.32E-05
NXPE4	21	19	2	1.660	13.620	0.996	1.018	1.109	0.415	3.961	3.73E-05
CPT1C	30	30	0	2.502	32.433	2.457	0.835	0.486	0.590	3.790	7.53E-05
KIAA1257	39	33	6	3.452	12.522	0.811	0.896	0.871	0.481	3.770	8.15E-05
NDUFA3	29	28	1	0.493	2.755	0.172	1.158	0.490	0.587	3.585	1.69E-04
EDNRB	15	13	1	1.797	14.472	1.186	0.806	0.329	0.410	3.523	2.14E-04
SGCA	21	21	0	1.209	16.236	0.808	0.878	0.420	0.620	3.497	2.35E-04
OGG1	27	21	6	1.732	13.857	1.345	1.103	0.885	0.576	3.473	2.58E-04
CDCA4	19	19	0	0.731	10.045	0.354	0.934	0.452	0.531	3.425	3.08E-04

Notes: Minor alleles refer to rare non-synonymous minor alleles in the tested cases. The minor allele counts to missense is summed in Missense alleles, and the minor allele counts to loss-of-function (stopgain, splicing, frameshift) is summed in LOF alleles. Region Length and ExonGC are the length (kb) and GC content in exon regions of the gene. mu_mis and mu_lof refer to the gene's mutation rate based on missense variants and loss-of-function variants in its canonical transcript, respectively. oe_mis and oe_lof refer to the gene's observed over expected ratio at missense and loss-of-function variants obtained from gnomAD, respectively. Residual and P mean deviance residual and *p*-value generated by RUNNER. $Residual_1$ and P_1 denote the deviance residual and *p*-value generated by RUNNER1 (the equal-weight (weight=1) version of RUNNER).

Table S10. Top genes of RUNNER with original and adjusted AD membership

	Original					Adjusted				
**Gene Symbol**	**Minor Alleles**	**Missense Alleles**	**LOF Alleles**	**$Residual_1$**	**P_1**	**Minor Alleles**	**Missense Alleles**	**LOF Alleles**	**$Residual_1$**	**P_1**
NAT1	27	27	0	5.159	1.24E-07	27	27	0	5.198	1.01E-07
PIF1	24	23	1	4.286	9.10E-06	23	22	1	4.223	1.20E-05
CXCL16	18	18	0	4.104	2.03E-05	16	16	0	3.807	7.02E-05

DPH1	21	18	3	4.035	2.73E-05	21	18	3	4.105	2.02E-05
PEX3	14	14	0	3.989	3.32E-05	11	11	0	3.364	3.84E-04
NIPAL1	16	16	0	3.922	4.39E-05	12	12	0	3.170	7.63E-04
CRK	11	11	0	3.836	6.24E-05	10	10	0	3.622	1.46E-04
ZNF814	25	6	19	3.733	9.46E-05	22	5	17	3.504	2.29E-04
ZNF479	22	9	13	3.661	1.26E-04	20	9	11	3.446	2.84E-04
IL10RA	19	19	0	3.655	1.29E-04	19	19	0	3.727	9.70E-05

Note: The AD membership was adjusted by age under a logistic regression model in the AD exome-sequenced sample. Subjects with the absolute value of deviance residual less than 0.8 will be ignored. In total, 20 patients were excluded. Minor alleles refer to rare non-synonymous minor alleles in the tested cases. The minor allele counts of missense were summed in Missense alleles, and the minor allele counts of loss-of-function (stop-gain, splicing, frameshift) were summed in LOF alleles. $Residual_1$ and P_1 denote the deviance residual and *p*-value generated by RUNNER1 (the equal-weight (weight=1) version of RUNNER).
